# CAR-CIK vs. CAR-T: benchmarking novel cytokine-induced killer cells as solid tumor immunotherapy in ErbB2+ rhabdomyosarcoma

**DOI:** 10.3389/fimmu.2025.1485817

**Published:** 2025-02-03

**Authors:** Laura M. Moser, Catrin Heim, Sebastian E. Koschade, Philipp Wendel, Süleyman Bozkurt, Sabine Harenkamp, Hermann Kreyenberg, Michael Merker, Christian Münch, Elise Gradhand, Meike Vogler, Evelyn Ullrich, Halvard Bönig, Jan-Henning Klusmann, Peter Bader, Winfried S. Wels, Eva Rettinger

**Affiliations:** ^1^ Division for Stem Cell Transplantation and Immunology, Department of Pediatrics, Goethe University Frankfurt, Frankfurt am Main, Germany; ^2^ Department of Pediatrics, Goethe University Frankfurt, Frankfurt am Main, Germany; ^3^ German Cancer Consortium (DKTK), partner site Frankfurt/Mainz, Frankfurt am Main, Germany; ^4^ Frankfurt Cancer Institute (FCI), Goethe University, Frankfurt am Main, Germany; ^5^ Universitäres Centrum für Tumorerkrankungen (UCT), Frankfurt am Main, Germany; ^6^ Department of Medicine, Hematology/Oncology, Goethe University Frankfurt, Frankfurt am Main, Germany; ^7^ Institute of Biochemistry II, Faculty of Medicine, Goethe University, Frankfurt am Main, Germany; ^8^ Experimental Immunology & Cell Therapy, Department of Pediatrics, Goethe University, Frankfurt am Main, Germany; ^9^ Institute for Organic Chemistry and Biochemistry, Technical University of Darmstadt, Darmstadt, Germany; ^10^ Department of Cellular Therapeutics/Cell Processing, Institute for Transfusion Medicine and Immunotherapy, Goethe University, Frankfurt am Main, Germany; ^11^ Cardio-Pulmonary Institute, Frankfurt am Main, Germany; ^12^ Department of Pediatric and Perinatal Pathology, Dr. Senckenberg Institute of Pathology, Goethe-University Frankfurt, Frankfurt am Main, Germany; ^13^ Institute for Experimental Pediatric Hematology and Oncology, Goethe University, Frankfurt am Main, Germany; ^14^ Division of Hematology, Department of Medicine, University of Washington, Seattle, WA, United States; ^15^ Georg-Speyer-Haus, Institute for Tumor Biology and Experimental Therapy, Frankfurt am Main, Germany

**Keywords:** cytokine-induced killer cells (CIK), CAR-T, rhabdomyosarcoma, solid tumors, ERBB2

## Abstract

**Introduction:**

CAR-T cell therapy, though successful in hematologic malignancies, faces challenges in solid tumors due to limitations of autologous T cells. Cytokine-induced killer (CIK) cells can be given safely across allogeneic barriers and constitute alternative effector cells generated from healthy donors. CIK cells are a heterogenous population of predominantly T cells with a mixed natural killer (NK) phenotype and combine non-MHC-restricted cytotoxicity with potent anti-tumor capacity of the adaptive immune system. Here, we characterize and compare efficacy, phenotypic subpopulations and modes of action of CAR-CIK cells and conventional CAR-T cells from same-donor samples in ErbB2+ rhabdomyosarcoma (RMS).

**Methods:**

To benchmark CAR-CIK against conventional CAR-T cells, effector cells were generated from same-donor samples and lentivirally transduced with a second generation CD28-CD3ζ CAR. Effector subpopulations and their dynamics upon target cell exposure were phenotypically characterized by flow cytometry. Efficacy was assessed in human ErbB2+ RMS cancer cell lines and primary patient samples *in vitro* and *ex vivo* using cytotoxicity and spheroid co-incubation assays. Modes of action were assessed by comparing cytokine secretion profiles using bead-based multiplexed flow cytometry and by liquid chromatography mass spectrometry whole cell proteomics. Finally, we used an *in vivo* model of RMS mimicking minimal metastatic residual disease to compare anti-tumor potency of CAR-CIK vs. CAR-T cells and to assess their target organ infiltration.

**Results:**

*In vitro* assays demonstrated superior cytotoxicity of CAR-CIK cells against RMS cell lines and primary tumor samples. Long-term co-incubation with tumor spheroids led to expansion of CAR-CIK cells and enrichment of CD3+CD56+ TNK cells. CAR-CIK cell cytokine signature showed significantly increased secretion of effector molecules like interferon-γ, perforin and granulysin, and lower secretion of Th2 cytokines IL-2, IL-4 and IL-10. Whole cell proteomics showed corresponding upregulation of chemokine signaling and NK-cytotoxicity pathways in CAR-CIK cells. In NSG mice xenografted with ErbB2+ RMS, a single injection of either CAR-effector cells strongly impeded metastatic tumor development and significantly improved survival.

**Conclusion:**

Our results demonstrate that CAR-CIK cells are at least equipotent to CAR-T cells. Combined with their favorable safety profile and allogeneic applicability, these findings position CAR-CIK cells as promising immune effectors for solid tumors.

## 1 Introduction

Adoptive cellular therapies have emerged as a powerful treatment option for hematological malignancies. In B cell neoplasias, chimeric antigen receptor (CAR) therapy has shown spectacular results and several CAR-T cell products have now been approved and adopted into standard therapy (1–5). However, the success of CAR-T cell therapy has so far been limited when applied to solid cancers (6). This can be attributed to several factors including antigen heterogeneity, antigen escape, high tumor burden, poor accessibility of solid tumors as well as an immunosuppressive tumor microenvironment (TME) (6–9). Low efficacy of CAR-T cell therapy can further be caused by fundamental limitations of autologous T cells (6, 10, 11). Immunosuppressive factors of the tumor can induce T cell dysfunction and (numerous) lines of previous therapy negatively affect the number of autologous T cells and their quality as starting material for CAR-T cell production. T cells from a (partially) human-leukocyte antigen (HLA)-matched healthy donor could overcome these issues but can be associated with lethal Graft-versus-Host Disease (GvHD) outside the setting of allogeneic hematopoietic stem cell transplantation (allo-HSCT) (10, 12). Although additional genetic manipulations can mitigate the risk of GvHD, they also generate higher costs, induce new genetic alterations and compromise T cell intrinsic tumor recognition via the T cell receptor (10). Alternative CAR-effector cells from healthy donors could help overcome these problems (9, 10, 12–14).

Cytokine-induced killer (CIK) cells have demonstrated efficacy and an excellent safety profile in clinical trials, even across HLA barriers in an allogeneic setting (14–20). They exert potent anti-tumor capacity against various hematological and solid malignancies (15, 21). CIK cells represent a heterogenous T cell population with a mixed natural killer (NK) cell phenotype and combine adaptive T cell-mediated with major-histocompatibility (MHC)-unrestricted activity of the innate immune system. While the CD3+CD56+ TNK cell subset within CIK cells shares some features with invariant NKT (iNKT) (20–24) and γδ T cells (25, 26), they are a distinct population with variable, mostly αβ T cell receptors (TCRs) arising from CD3+ T cells (27–35). CIK cells are easily generated from peripheral blood mononuclear cells (PBMCs), expanded by *in vitro* cytokine addition and can be successfully engineered to express CARs (14, 15, 18, 36–38). However, these novel effector cells have not yet been directly benchmarked against conventional CAR-T cells, and their comparative efficacy remains unknown.

We chose advanced pediatric rhabdomyosarcoma (RMS) as a solid tumor model to comparatively assess CIK versus conventional T cells for CAR-engineering. In RMS, new treatment options and medical advances have not translated into better outcomes and there is a high medical need to develop alternative therapies (39). Several types of sarcoma express ErbB2 (HER2) at low levels, rendering this receptor tyrosine kinase a potential target for immunotherapy (40). First clinical evaluations of ErbB2-directed CAR-T cells for sarcomas have proven the safety and feasibility of this approach but had limited efficacy (41–43).

We have previously shown that ErbB2-specific CAR-CIK cells mediate strong and specific activity against RMS (34, 44). Here, we present the first comprehensive comparative efficacy analysis *in vitro* and *in vivo* of CAR-CIK versus CAR-T cells with equal CAR-expression from same-donor samples targeting ErbB2 in RMS, including a thorough phenotypic characterization of these distinct CAR-effector cells. Using established RMS cell lines and primary samples from patients with metastatic disease, we demonstrate increased cytolytic activity of CAR-CIK cells *in vitro* and equipotency *in vivo*. Assessment of cytokine signatures and whole cell proteomics uncovered marked differences in the mechanisms of action of CAR-T and CAR-CIK cells, with the latter exerting their effects predominantly through NK cell-like cytotoxic pathways and related effector molecules. Given the limitations of autologous CAR-T cells, these findings position CAR-CIK cells as promising immune effectors for new cellular therapies in solid tumors.

## 2 Materials and methods

### 2.1 Primary RMS

Primary RMS samples were obtained from two pediatric RMS patients after written informed consent of their legal guardians in accordance with the Declaration of Helsinki. The ethics review board of the Goethe University (Frankfurt, Germany) approved the biomaterial usage (SPO-04-2015). H1 samples were from a cutaneous lesion of a progressing alveolar Pax3-FOXO1 fusion-positive RMS, H9 samples were from pleural metastases of an embryonal fusion-negative RMS. Both primary RMS samples were cultivated in DMEMHighGlucoseHepesGlutaMAX (ThermoFisher) supplemented with 10% FBS, 1% Penicillin/Streptomycin and 1% sodium-pyruvate.

### 2.2 Lentiviral particles and transduction of cell lines

GFP/luciferase-expressing as well as mCherry/luciferase-expressing cells were produced by lentiviral transduction using the pSIEW-luc2 (45) and pLenti_fLuc_mCherry plasmids and were enriched by fluorescence-activated cell sorting (sorting for GFP+ or mCherry+ cells, respectively) using a FACSAria II instrument (BD Biosciences).

### 2.3 Generation of parental and ErbB2-CAR-CIK and CAR-T cells

PBMCs from healthy volunteer donors were used as a source for CIK and T cells with a minimum of three independent donors for all *in vitro* experiments (range 3–5). PBMCs were collected via standard density gradient separation using Histopaque-1077 (Sigma-Aldrich) after informed consent had been provided. The study and biomaterial usage were approved by the Ethics Review Board of the Medical Faculty of the University Hospital Frankfurt (Main), Germany (Nr. 413/15). T cells were isolated from PBMCs using the EasySep™ Human T cell isolation kit (StemCell) according to the manufacturer’s instructions and cultivated in RPMI 1640 medium with GlutaMAX with 10% FBS, 2 mM L-Glutamine (ThermoFisher), 25 mM Hepes (ThermoFisher) and 50 µM 2-mercapto-ethanol containing 100 U/mL human IL-2 (Novartis). T cells were seeded in 48-well plates and stimulated with 25 µL/mL ImmunoCult Human CD3/CD28 T-Cell Activator (StemCell) on day 0. Cell density was adjusted to 1–2x10^6^ cells/mL with new culture medium containing 100 U/mL IL-2 every 2–3 days.

CIK cells were cultivated as previously described (46): In brief, cells were resuspended at a density of 3x10^6^ cells/mL in RPMI 1640 with GlutaMAX and 10% FBS in 6-well plates and primed by adding 1000 U/mL Interferon-γ (IFNγ, Boehringer Ingelheim) on day 0. 100 ng/mL anti-CD3 antibody (MACS GMP CD3 pure, Miltenyi Biotech) and 500 U/mL IL-2 were added after 24 h. 50 ng/mL human IL-15 (Peprotech) was added on day 3. Over the course of the culture, cell density was adjusted to 1x10^6^ cells/mL, fresh medium and 50 ng/mL IL-15 were added on day 5 and 8.

To generate ErbB2-CAR-CIK and CAR-T cells, transduction with lentiviral particles (produced using the pS-5.28.z-IEW transfer plasmid) was performed via spinfection on day 2 of culture. The p.S-5.28z-IEW vector was described previously (34, 44, 47): it encodes a codon-optimized second-generation CAR with the ErbB2-specific scFv (FRP5) antibody fragment, modified CD8α hinge region, CD28 transmembrane and intracellular domains and a CD3ζ intracellular domain inserted upstream of IRES and eGFP sequences of the pSIEW vector backbone (48). eGFP was used as a fluorescence marker. Transduction protocols were optimized to achieve comparable CAR-expression in CAR-CIK and CAR-T cells. For T cells, the cell number was adjusted to 0.25x10^6^ cells/well, 8 µg/mL polybrene (Sigma-Aldrich) and 100 µL viral particles were added and volume was topped up to 1 mL/well with culture medium. For CIK cells, the cell number was adjusted to 0.5x10^6^ cells/well, 8 µg/mL polybrene and 1000 µL viral particles were added and volume was topped up to 2 mL/well with culture medium. Spinfection was done at 800 g (acceleration 4/10, no brake) for 50 minutes at 32°C. Afterwards, half of the culture medium was exchanged with fresh culture medium containing IL-2 (100 U/mL) for transduced T cells, whereas transduced CIK cells were incubated for 24 hours and then stimulated with 50 ng/mL IL-15. T cell density was adjusted to 1–2x10^6^ cells/mL with new culture medium containing 100 U/mL IL-2 every second or third day. CIK cell density was adjusted to 1x10^6^ cells/mL at day 5 and 8 of culture and fresh culture medium and 50 ng/mL IL-15 were added. All cells were expanded over 10 days and then used for further analyses. Effector cell numbers utilized in experiments encompass the entire population without specific selection for CAR+ cells, ensuring representation of the heterogeneous CIK cell population, including NK and TNK cells transduced at a lower rate. Transduction methods were optimized to ensure equal CAR-expression rates in bulk CAR-CIK and CAR-T cells across all analyses.

### 2.4 Luciferase toxicity assay

RMS cells stably expressing firefly luciferase (fLuc) were used for 24 h toxicity assays as previously described (49). Effector cells were added at effector to target (E:T) ratios from 10:1 to 1.25:1. Results are given as percentage of luciferase signal of untreated target cells.

### 2.5 Spheroid co-incubation assay

For generation of Rh30fLuc/mCherry and Rh41fLuc/mCherry spheroids, 5x10^3^ cells were seeded in 200 µl of RPMI with 10% FBS into ultralow attachment 96-well round-bottom plates without prior coating (Corning). After 4 days, 100 μL of supernatant was removed, and 25x10^3^, 12.5 x10^3^ or 6.25 x10^3^ effector cells resuspended in 100 μL medium (without cytokines) were added. Spheroids without effector cells were used as controls. Experiments were set up in duplicates. Co-cultures were incubated at 37°C and imaged every 8 hours using the Incucyte S3 System (Incucyte 202B, Sartorius) for 6 days. Half of the medium was exchanged after 3 days (no additional cytokines). The spheroid feature of the Incucyte S3 software was used to quantify growth of mCherry+ spheroids by measuring the integrated red intensity over time. After co-incubation with Rh30fLuc/mCherry spheroids, effector cells were analyzed via flow cytometry.

### 2.6 Whole cell proteomics

For whole cell proteomics, CAR-CIK, CAR-T and untransduced parental cells were co-incubated ± Rh30 cells at an E:T ratio of 2:1. After 24 hours, CD45+ effector cells were isolated via FACS (CD45 PacificBlue, HI30, Biolegend) using a BD FACS Aria 3, collected into 1.5 mL tubes (Eppendorf), spun down and cryopreserved. Sample preparation, fractionation and liquid chromatography mass spectrometry was performed as described in the **Supplementary Methods**. As no relevant differences were observed for the condition ± RMS, results were aggregated for each effector cell. For data analysis and plotting, R version 4.2.2 was used together with data.table 1.14.10 and ggplot2 3.4.4. Differential expression changes comparing CAR-CIK vs. CAR-T cells were analyzed using DEqMS 1.16.0. For KEGG pathway analysis, the list of all quantified proteins was ranked (absolute log2 fold chance x –log10-transformed q-value) and gene set enrichment analysis was done with clusterProfiler version 4.6.2 using default settings and FDR-correction of P values. The mass spectrometry proteomics data have been deposited to the ProteomeXchange Consortium via the PRIDE partner repository (50) with the dataset identifier PXD050654.

### 2.7 *In vivo* metastatic RMS xenograft model

Female non-obese diabetic (NOD)/severe combined immunodeficient (SCID)/IL-2receptorγ−/− (NSG) mice were used (age 6–12 weeks). To mimic the clinical situation of residual RMS after heavy pretreatment (44), mice were sublethally irradiated with 2.5 Gy (Biobeam 2000) on day -1. One day later (d0), 1x10^5^ Rh30GFP/fLuc+ cells resuspended in 100 μL of PBS were injected via the tail vein, allowing for metastatic tumor spread into liver, lungs and bone marrow. Mice were randomly divided into five different groups: control animals (n=7) received 100 µL medium (GlutaMAX with 10% FBS) and treatment groups (CAR-CIK, CAR-T, CIK, T) received 2.5x10^6^ effector cells resuspended in 100 µL medium via tail vein injection on day +1. Group sizes were predetermined based on prior data (n=10–12 mice for CAR-engineered cells; n=5–6 for parental cells; ≥ 5 animals with vehicle control). Tumor growth was monitored weekly by bioluminescence imaging (BLI) using an IVIS Lumina II system (Perkin Elmer). Mice were anesthetized via isoflurane inhalation and 1500 μg *in vivo* grade VivoGlo luciferin (Promega) in 100 μL PBS was injected subcutaneously. After 15 min, images were acquired at serial exposure times (1 s–4 min) and analyzed with Aura Spectral Instruments imaging software version 2.7.11. Photon flux was quantified per mouse in uniform regions of interest.

Animal experiments were approved by the responsible government oversight committee (Regierungspräsidium Darmstadt, Dezernat V54, ref. FK/2033). All applicable guidelines for housing, care and use of animals were followed. Mice were inspected minimum once daily, assessed and scored for disease activity; moribund mice were sacrificed. Peripheral blood, bone marrow (BM), lung, liver and spleen samples were isolated and analyzed for persistence of human tumor or effector cells (49): In brief, organs were cut in half and partly conserved in formaldehyde for immunohistochemistry. The remaining organ tissue was processed into single cell suspensions and analyzed via flow cytometry for human CD45+ effector cells.

### 2.8 Supplementary methods

A detailed description of cell lines, flowcytometry staining and antibodies, the detection of immune effector molecules via bead-based immuno-assay, immunohistochemistry of mouse organs, histology of primary patient material as well as a more detailed description of lentiviral particle production, transduction of cell lines, luciferase-based toxicity assays, whole cell proteomics and animal experiment reporting can be found in the **Supplementary Material**.

### 2.9 Statistics

For statistical analysis and plotting, GraphPadPrism 10.2.0, R 4.2.2, data.table 1.14.10, ggplot2 3.4.4 and DEqMS 1.16.0 were used. Unless otherwise indicated in the figure legends, data are displayed as arithmetic mean ± standard error of the mean (SEM). The exact sample size for each experimental group or condition is given in the figure legends. Only biological replicates are shown and used for statistical inferences. Where applicable, paired t-tests were used, otherwise the statistical method is described in the figure legend. Survival analyses were performed using the Kaplan-Meier method and Log-rank (Mantel cox) tests to compare treatment-groups. Group differences with p < 0.05 (*), p < 0.01 (**) or p < 0.001 (***) were considered statistically significant.

## 3 Results

### 3.1 ErbB2-CAR CIK cells are easily produced by lentiviral transduction and maintain their distinctive CIK cell phenotype of TNK cells

**Figure 1:**
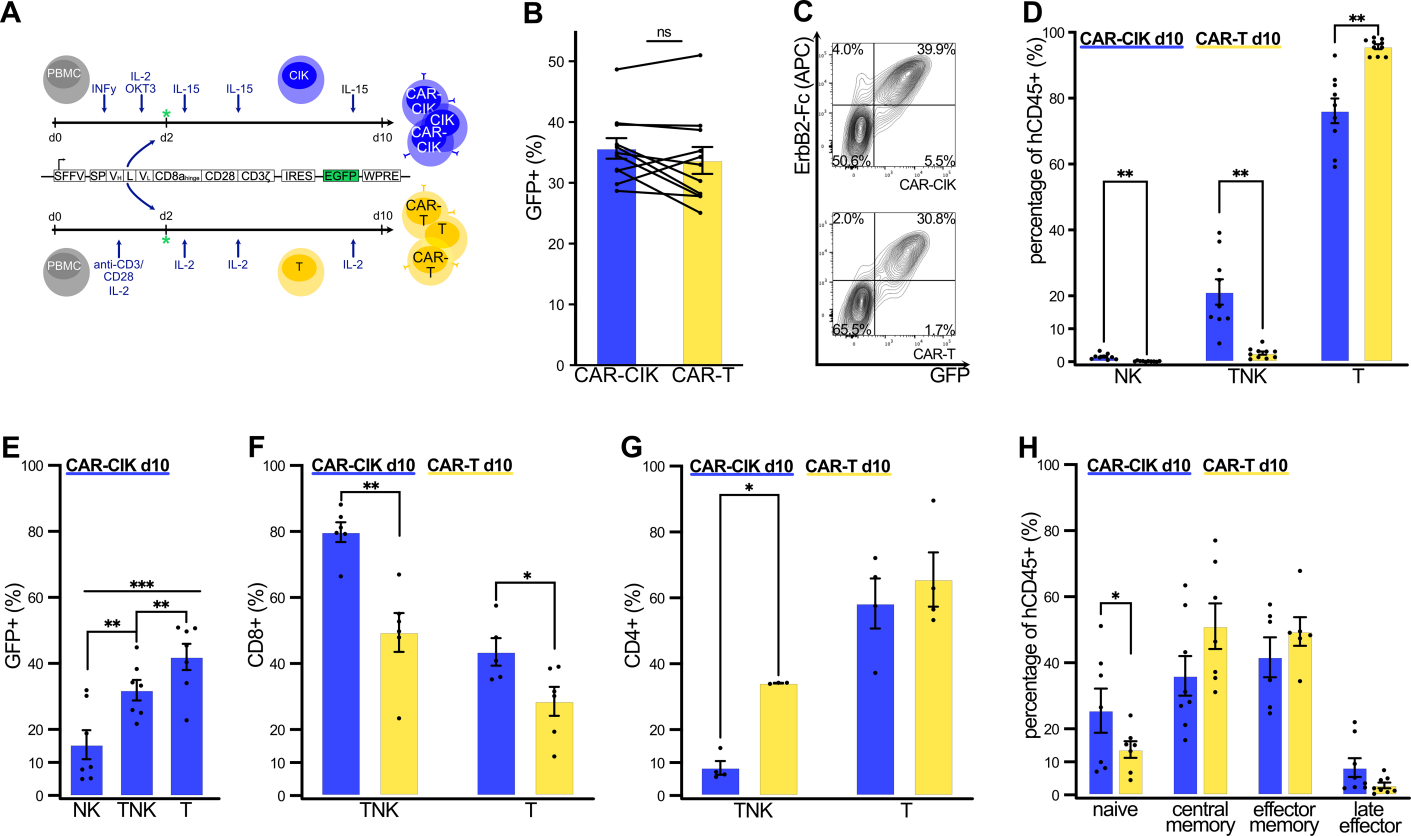
**(A)** Cultivation and transduction of CAR-CIK and CAR-T cells with a second-generation ErbB2-specific CD28-CD3ζ CAR. All endpoint phenotypic characterizations were performed by flow cytometry on day +10 of *in vitro* expansion. **(B)** Transduction rates of CAR-CIK and CAR-T cells, measured by GFP expression. Mean ± SEM of n=11. **(C)** Representative flow cytometry plots showing double expression of GFP and ErbB2-CAR in CAR-CIK and CAR-T cells. **(D)** CD3 and CD56 expression by CAR-CIK and CAR-T cells, identifying NK (CD3-CD56+), TNK (CD3+CD56+) and T cell subpopulations (CD3+CD56-). Mean ± SEM of n=9–10. **(E)** Transduction rates in the NK, TNK and T cell compartments of CAR-CIK cells. Mean ± SEM of n=7. **(F)** CD8+ phenotype of TNK cells and T cells in CAR-CIK and CAR-T cells. Mean ± SEM of n=5–6. **(G)** CD4+ phenotype of TNK cells and T cells in CAR-CIK and CAR-T cells. Mean ± SEM of n=4. **(H)** Subpopulations of naive (CD62L+CD45RO-), central memory (CD62L+CD45RO+), effector memory (CD62L-CD45RO+) and late effector (CD62L-CD45RO-) cells in CAR-CIK and CAR-T cells. Mean ± SEM of n=6–8 experiments. P values calculated by paired t-tests. Group differences with p < 0.05 (*), p < 0.01 (**) or p < 0.001 (***) were considered statistically significant.

To enable direct comparison, CIK and T cells were derived in parallel from same-donor PBMCs with a minimum of three independent donors (**Figure 1A**). Lentiviral transduction on day 2 of culture resulted in robust and equal CAR-expression in both CAR-CIK and CAR-T cells (GFP+ in CAR-CIK 35.7% ± 1.7% vs. 33.7% ± 2.2% in CAR-T, not significant) (**Figures 1B, C**, **Supplementary Figure 1**). CAR-CIK cells developed a mixed double-positive TNK population, with 21.2% (± 3.9%) of cells expressing both CD3 and CD56. The TNK population was negligible among CAR-T cells (**Figure 1D**). Transduction was most efficient in the CD3+CD56- T cell and the CD3+CD56+ TNK cell subpopulation of CAR-CIK cells and was seen to some extent in the CD3-CD56+ NK cell compartment of CAR-CIK cells (**Figure 1E**). CAR-CIK cells showed a predominant CD8+ cytotoxic phenotype, whereas CAR-T cells showed a CD4+ helper T cell phenotype (**Figures 1F, G**). *In vitro* expansion led to a reduction in naïve cells among both effector cell populations and to a shift towards a central memory and effector memory phenotype, with a higher percentage of naïve cells seen among CAR-CIK compared to CAR-T cells (**Figure 1H**, **Supplementary Figure 1**). Both transduced cell populations displayed high viability and equal expansion over the course of 10 days in culture (**Supplementary Figure 1**). Thus, CAR-CIK and CAR-T cells can be generated easily by lentiviral transduction at equal transduction rates, with CAR-CIK cells developing the distinctive TNK and effector phenotype of CIK cells.

### 3.2 ErbB2-CAR CIK cells show superior *in vitro* cytotoxicity

**Figure 2:**
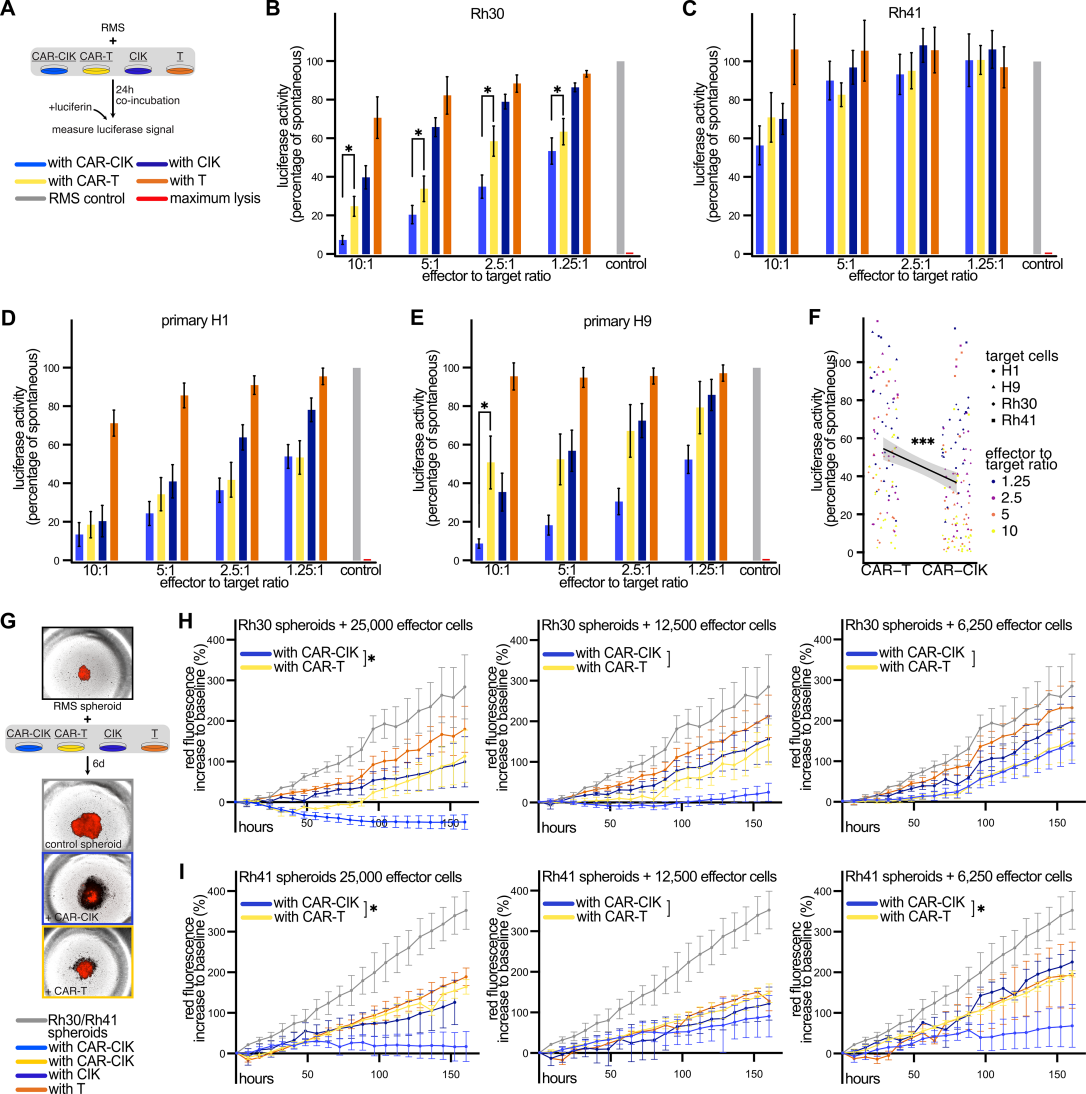
**(A)** Experimental scheme. Co-incubation of parental and ErbB2-CAR-CIK and CAR-T cells and T cells with luciferase-expressing target cells for 24 h at different effector to target ratios. Results are given as percentage of luciferase signal of untreated target cells. TritonX-100-treated cells served as maximum lysis control. **(B–E)** Cytotoxicity against B) Rh30 (n=8–10), **(C)** Rh41 (n=3–4), and against primary patient-derived tumor cells **(D)** H1 (n=6–9) and **(E)** H9 (n=6–7). Mean ± SEM are shown. P values calculated by Wilcoxon tests comparing CAR-CIK vs. CAR-T cells. **(F)** A linear regression model was used to compare the effect of CAR-CIK vs. CAR-T cells across all four target cells and all effector to target ratios with these parameters as independent predictors. Individual values and regression line with 95% confidence interval (shaded grey area) are shown. **(G)** Experimental scheme with representative microscopy images. **(H, I)** Parental and CAR effector cells were co-incubated for 6 d with established tumor spheroids generated from Rh30mCherry (**H**, n=5) or Rh41mCherry cells (**I**, n=3) and monitored via fluorescence microscopy. Difference to baseline (1 h after effector cell addition) of red fluorescence of the tumor spheroids is given in percent. Mean ± SEM are shown. P values by unpaired t-tests, comparing areas under the curves (AUC). Group differences with p < 0.05 (*), p < 0.001 (***) were considered statistically significant.

In 2D *in vitro* co-cultures with RMS cell lines and primary patient material (**Figure 2A**), we observed potent anti-tumor activity of CAR-CIK and CAR-T cells in a dose-dependent manner. Against the alveolar RMS cell line Rh30, CAR-CIK cells proved more efficacious than CAR-T cells at all effector to target (E:T) ratios (**Figure 2B**). For the TP53-mutated alveolar RMS cell line Rh41, we observed a less pronounced yet equally potent cytotoxicity of CAR-CIK and CAR-T cells (**Figure 2C**). Next, we tested both CAR-effector cells against primary tumor samples (H1, H9) obtained from relapsed, metastatic pediatric RMS that were confirmed to express ErbB2 via flow cytometry (**Supplementary Figure 2**). We observed a clear trend towards superior cytotoxicity of CAR-CIK cells compared to CAR-T cells, as well as dose-dependent increased efficacy at higher E:T ratios (**Figures 2D, E**). Overall, a linear regression model across all target cell types and E:T ratios confirmed significantly higher killing of CAR-CIK compared to CAR-T cells (**Figure 2F**).

To further confirm the potency of CAR-CIK cells in 3D models, we generated RMS tumor spheroids and performed long-term co-incubation of the CAR effector cells with established tumor spheroids (**Figure 2G**). Over the course of 6 days, both CAR-effector cells potently inhibited spheroid growth and even led to lysis of Rh30 tumor spheroids at higher concentrations, with CAR-CIK cells proving more potent than CAR-T cells at the highest E:T ratio (**Figure 2H**). Against Rh41 spheroids, CAR-CIK proved more efficacious than CAR-T cells at two out of three assessed effector concentrations (**Figure 2I**). Taken together, these results show superior killing activity of CAR-CIK vs. CAR-T cells in multiple *in vitro* models of RMS.

### 3.3 CAR-CIK cells proliferate and show enrichment of CAR+ and TNK cells upon target exposure

**Figure 3:**
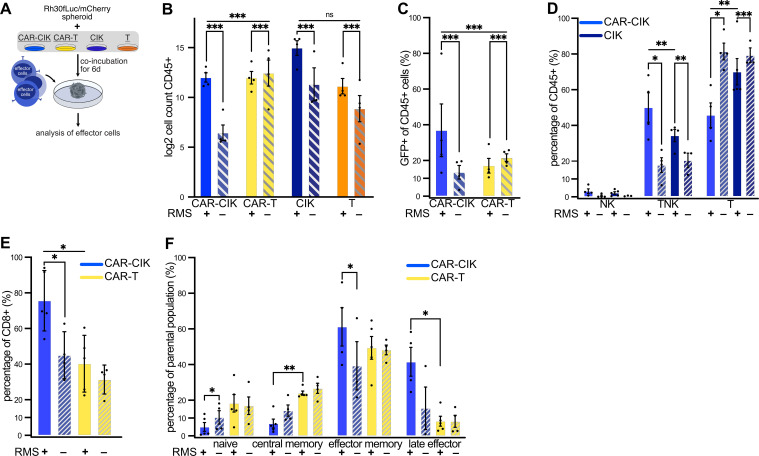
**(A)** Experimental scheme. Flow cytometric analysis of effector cells after 6 d co-incubation of parental and ErbB2-CAR-CIK and ErbB2-CAR-T cells with Rh30 spheroids. **(B)** Effector cell counts (log2-transformed) after 6 d ± RMS spheroids. Mean ± SEM of n=3–4; comparison of ± RMS by Poisson regression on untransformed count data; between-group effect of ± RMS was compared between CAR-CIK and CAR-T by testing for differences in slope of Poisson regression lines (likelihood ratio test). **(C)** Percentage of GFP+ cells after 6 d ± RMS. Mean ± SEM of n=4; analysis by Poisson regression as done in **(B, D)** CD3 and CD56 expression of CAR-CIK and CIK cells after 6 d ± RMS given as percentages of NK cells (CD3-CD56+), TNK cells (CD3+CD56+) and T cells (CD3+CD56-). Mean ± SEM of n=3–5, comparing CAR-CIK + RMS vs. CIK + RMS, CAR-CIK + vs. – RMS and CIK + vs. – RMS. P values by paired t-tests. **(E)** Relative CD8 expression of CAR-CIK and CAR-T cells after 6 d ± RMS. **(F)** Relative sizes of naive (CD62L+CD45RO-), central memory (CD62L+CD45RO+), effector memory (CD62L-CD45RO+) and terminal effector (CD62L-CD45RO-) subpopulations after 6 d ± RMS of CAR-CIK and CAR-T cells. Mean ± SEM of n=3–4 **(E)** and n=3–5 **(F)**. P values by paired t-tests, comparing CAR-CIK + RMS vs. CAR-T + RMS, CAR-CIK + vs. – RMS and CAR-T + vs. – RMS, respectively. Group differences with p < 0.05 (*), p < 0.01 (**) or p < 0.001 (***) were considered statistically significant.

Analysis of effector cells after long term co-incubation with Rh30 tumor spheroids over 6 days without additional cytokine stimulation (**Figure 3A**) revealed that CAR-CIK cells proliferated upon target exposure, whereas CAR-T cells did not (**Figure 3B**). The GFP+ population (GFP being co-expressed with the CAR) was significantly enriched only in CAR-CIK cells after spheroid co-incubation, indicating preferential expansion of CAR-bearing CIK cells (**Figure 3C**). Furthermore, prolonged co-culture with RMS spheroids led to an increase of the CD3+CD56+ TNK cell compartment of CAR-CIK cells (**Figure 3D**). After co-culture, CAR-CIK cells showed a predominant CD8+ phenotype, while there was no significant change in the CD8+ phenotype of CAR-T cells (**Figure 3E**). Likewise, the memory/naïve phenotype of CAR-T cells did not change appreciably after co-culture (**Figure 3F**). By contrast, CAR-CIK cells displayed a considerable change, with fewer naïve cells and more effector memory cells. CAR-CIK cells co-incubated with RMS spheroids also contained a smaller population of central memory and a higher proportion of terminal effector cells than CAR-T cells (**Figure 3F**). These results show that CAR-CIK cells exhibit clear phenotypical changes upon RMS target contact with preferential expansion of CAR-bearing cells and acquisition of effector cell characteristics, unlike CAR-T cells.

### 3.4 CAR-CIK cells show NK cell cytotoxicity *in vitro*

**Figure 4:**
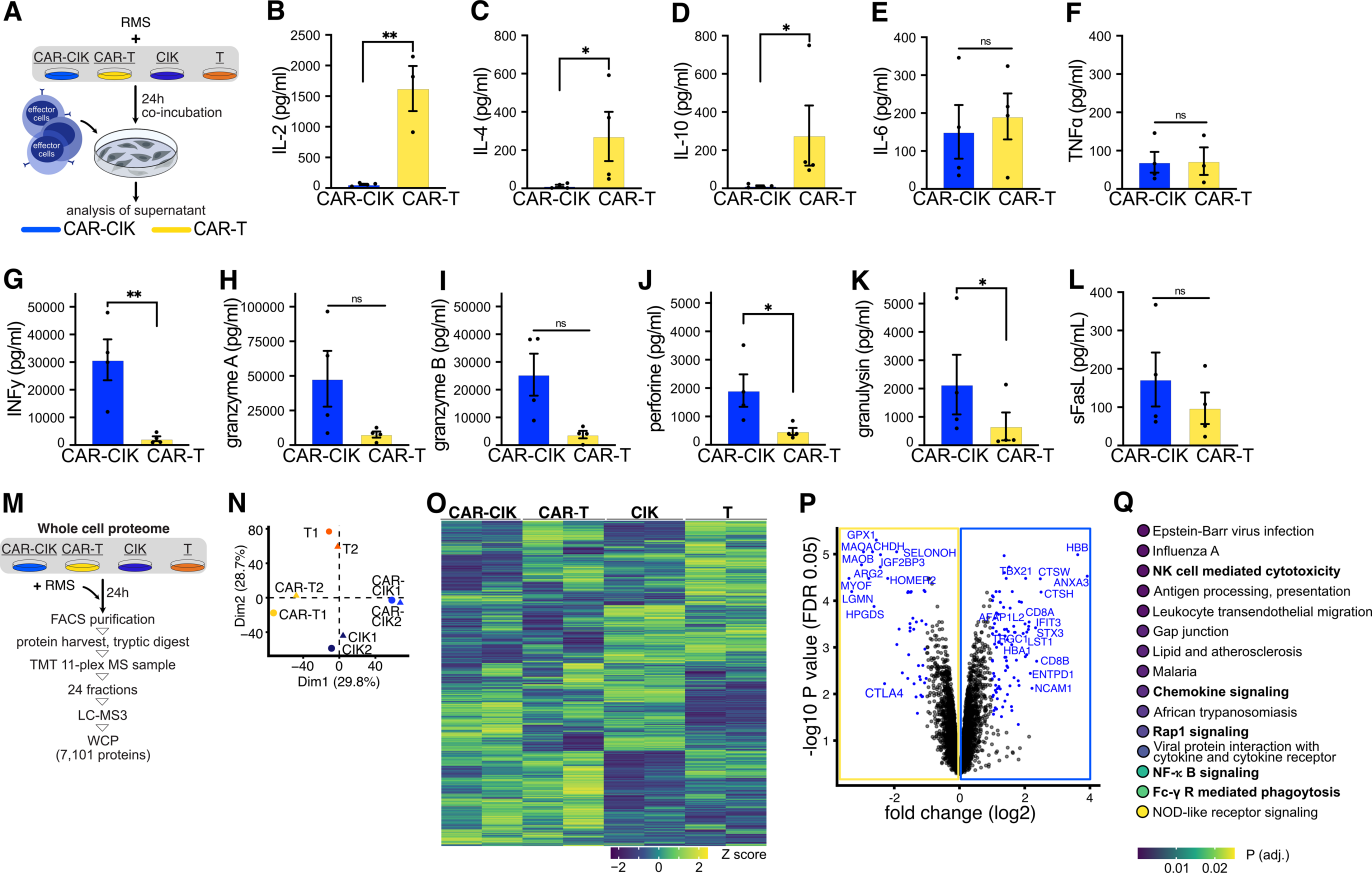
**(A–L)** After 24 h co-incubation with Rh30 cells (effector to target ratio of 10:1), supernatants of CAR-CIK or CAR-T cells were harvested and quantitatively analyzed for their cytokine signature using a bead-based immuno-assay. Mean ± SEM of n=3–4. P values were obtained by testing for differences in log2-transformed concentrations (pg/mL) by paired t-tests. sFasL, soluble Fas-Ligand. **(M)** Experimental scheme. Effector cells were FACS-purified and whole-cell proteomics (WCP) was performed using liquid chromatography mass spectrometry (LC-MS). TMT, tandem mass tag. **(N)** Principal component analysis of all conditions and replicates. Dim, dimensions. **(O)** Heat map showing row-scaled Z scores for all measured proteins across all conditions. **(P)** Volcano plot depicting log2 fold changes (FC) in measured proteins and false discovery rate (FDR)-corrected P values (q values; DEqMS/limma-moderated two-sided t-test). Significantly changing (q < 0.05) proteins with an absolute log2 FC ≥ 1 are highlighted in blue, proteins with an absolute log2 FC ≥ 2 are labeled. **(Q)** KEGG gene set enrichment analysis on full ranked data from **(P)**; the top 15 significantly enriched pathways are shown. Group differences with p < 0.05 (*), p < 0.01 (**) were considered statistically significant.

We next sought to analyze modes of action of CAR-CIK and CAR-T cells (**Figure 4A**). After 24 h co-incubation with Rh30 cells, cytokine profiles in the supernatants of CAR-CIK and CAR-T cells differed profoundly. Whereas CAR-T showed high secretion of IL-2, IL-4 and IL-10, which had a very low concentration in CAR-CIK conditioned media, CAR-CIK supernatants contained high levels of effector cytokines and direct mediators of cytotoxicity, such as IFNγ, granulysin, perforin, granzymes and soluble Fas-Ligand (sFasL). IL-6 and TNFα were found at similar levels in both conditions (**Figures 4B–L**).

To identify potential differences in CAR-CIK and CAR-T cells responsible for these contrasting cytokine secretion profiles, we performed whole cell proteomics and measured differentially expressed proteins in CAR-CIK and CAR-T cells as well as untransduced parental CIK and T cells. Cells were FACS-purified and prepared for liquid chromatography mass-spectrometry using tandem-mass tag (TMT)-based multiplexing and offline fractionation. This approach quantified 7101 distinct proteins (**Figure 4M**). Unsupervised clustering showed excellent separation in protein expression according to cell type and transduction status (**Figures 4N, O**). Proteins significantly depleted in CAR-CIK cells compared to CAR-T cells were negative regulators of T cell activation (HOMER2, CTLA4), proteins expressed in Th2 cells (HPGDS, GPX1) and during CD4+ activation (LGMN) as well as proteins negatively influencing T cell function (ARG2, MAO-A). Proteins found to be significantly enriched in CAR-CIK cells pertained to NK cell differentiation (NCAM1/CD56), TNK cell differentiation (TRGC1), CD8 (CD8B, CD8A) and Th1 differentiation (ANXA3), INFy response (TBX21, HBB), vesicle formation (STX3, ANXA3), granzyme B secretion (CTSH, TBX21), activated (NK) cells (ENTPD1, AFAP1L2) and cellular cytotoxicity of NK cells (CTSW, TBX21) (**Figure 4P**, **Supplementary Table 1**). Finally, unbiased KEGG pathway analysis of whole cell proteomics data revealed NK cell mediated cytotoxicity, chemokine signaling, Rap1 signaling (mediating NKG2D-driven cytotoxicity) (51), NF-κB signaling and Fc-γ receptor signaling as some of the most relevant pathways overexpressed in CAR-CIK cells compared to CAR-T cells (**Figure 4Q**). These profound mechanistic differences strongly suggest that CAR-CIK cells, despite their CD3+ T cell majority and the CAR-transduction, act like NK cells in conveying their cytotoxicity.

### 3.5 CAR-CIK effector cells demonstrate equal potency in an *in vivo* metastatic RMS xenograft model

**Figure 5:**
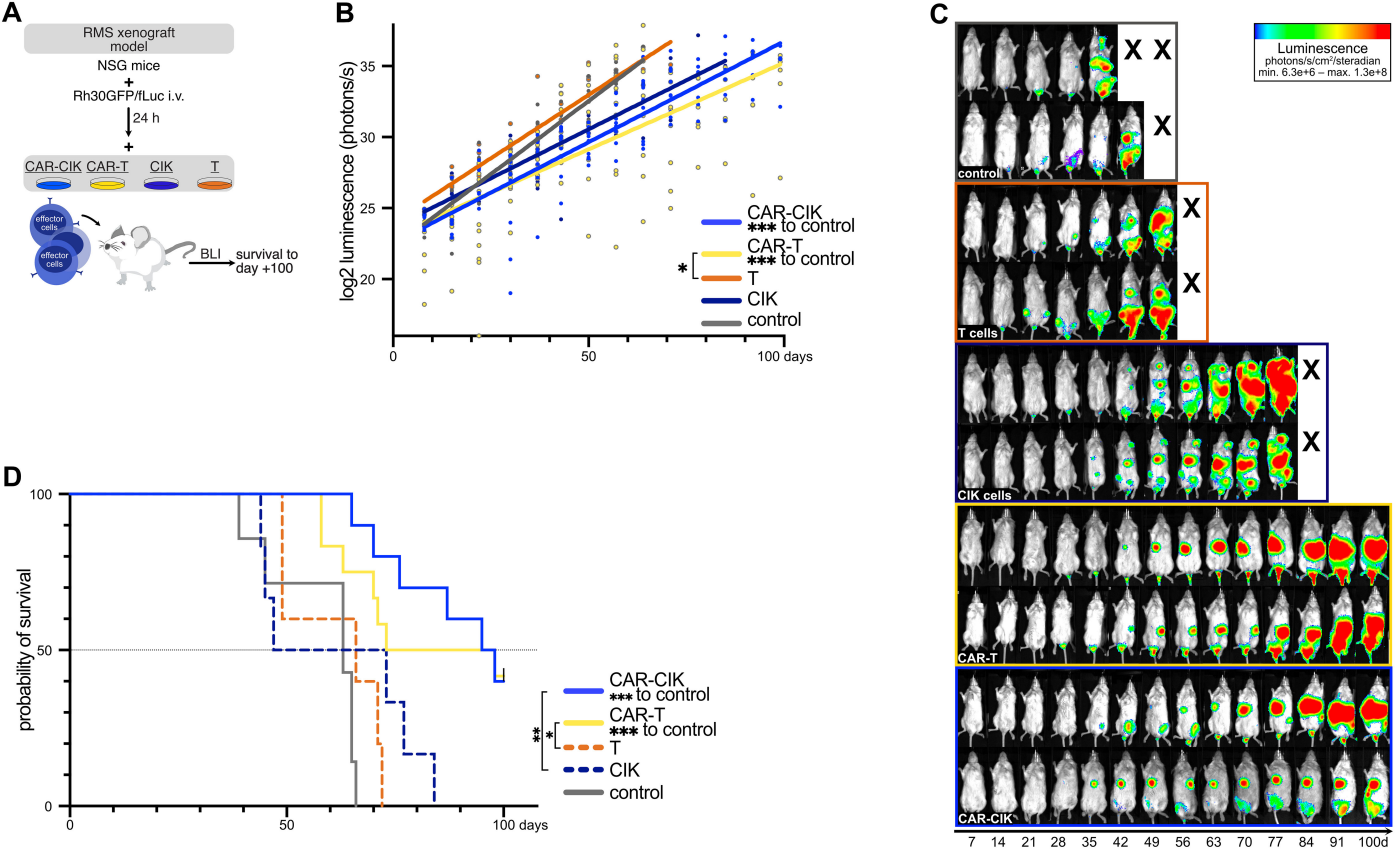
**(A)** Schematic overview of the *in vivo* RMS xenograft model. After sublethal irradiation on day -1, luciferase-expressing Rh30 cells (Rh30/GFPfLuc) were transplanted into NSG mice by intravenous injection. After 24 h, mice were injected with CAR-effector cells all generated from the same-donor sample (CAR-CIK, n=10; CAR-T, n=12) or the parental, untransduced populations of CIK cells (n=6) or T cells (n=5). Control mice (n=7) received vehicle control. **(B)** Quantification of serial ventral luminescence measurements after treatment. Log2-transformed individual measurements and group-wise linear regressions are shown. P values by linear regression, comparing slope coefficients between groups. **(C)** Serial bioluminescence (BLI) images of two representative animals of each group on the indicated days after transplantation; luminescence displayed as log2-transformed photons/s/cm^2^/steradian. **(D)** Overall survival of xenografted NSG mice treated with vehicle, CAR-engineered or parental effector cells. P values for the differences in survival were obtained by log-rank tests.

We next wanted to compare the *in vivo* efficacy of the CAR-effector cells using a metastatic RMS xenograft model mimicking minimal residual disease. After sublethal irradiation, NSG mice received luciferase-expressing RMS tumor cells (Rh30GFP/fLuc) via tail vein injection. 24 h later, mice were randomized into five experimental groups and treated with (1) 2.5x10^6^ CAR-CIK cells, (2) 2.5x10^6^ CAR-T cells (with equal CAR-expression, **Supplementary Figure 3B**), (3) 2.5x10^6^ parental CIK cells, (4) 2.5x10^6^ parental T cells, or (5) vehicle control. Tumor burden and disease progression were assessed by weekly bioluminescence imaging (BLI) and clinical scoring for a period of 100 days (**Figure 5A**). Every animal developed tumors detectable via BLI. CAR-CIK- and CAR-T-treated mice showed significantly lower BLI tumor burden than controls (**Figures 5B, C**). Tumor growth progressed most rapidly in vehicle control mice, with a median survival of 63 days (range, 39–66 days; **Figure 5D**). Neither untransduced CIK nor T cells significantly slowed tumor growth in comparison to controls (CIK cells: median survival 60 days, range 45–84 days; T cells: median survival 66 days, range 49–72 days; **Figure 5D**). However, the single application of either CAR-CIK or CAR-T cells markedly delayed metastatic tumor development and significantly improved the duration of survival compared to parental immune effector cells or controls. CAR-T-treated animals showed a median survival of 86 days (range 58–100 days), while CAR-CIK-treated animals showed a median survival of 97 days (range 65–100 days). Thus, the metastatic RMS model showed equipotency of CAR-CIK cells *in vivo*.

### 3.6 ErbB2-CAR-CIK cells migrate to tumor-infiltrated organs and display a predominant effector phenotype *in vivo*

**Figure 6:**
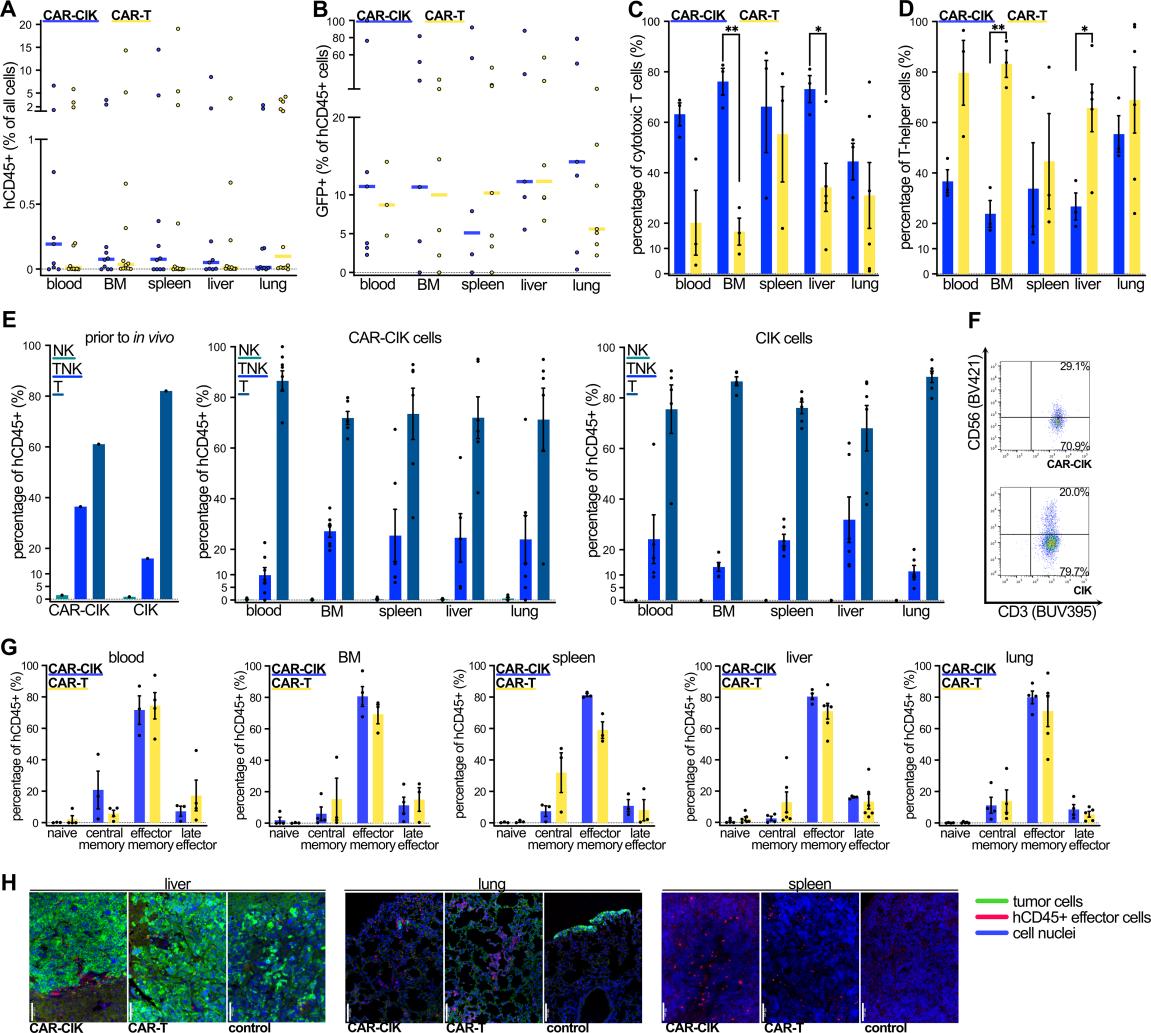
Flow cytometric analysis of cells from blood, bone marrow (BM), spleen, liver and lungs of CAR-CIK- and CAR-T-treated animals at the end of the experiment. **(A)** Median and individual values of human CD45+ (hCD45+) cells as percentage of total analyzed cells for n=8–12. **(B)** Median and individual values of GFP+ cells as percentage of hCD45+ cells for n=5–7. **(C)** Phenotype of cytotoxic (CD8+) and **(D)** T-helper cells (CD8-) of CAR-CIK and CAR-T cells retrieved from indicated organs. Mean ± SEM of n=3–6. P values by unpaired t-tests. **(E)** TNK cell phenotype according to CD3 and CD56 expression of CAR-CIK and CIK cells prior to injection and of cells retrieved from indicated organs. Mean ± SEM of n=4–6. **(F)** Representative flow cytometry plot of CD56 and CD3 expression on CAR-CIK and CIK cells from the spleen. **(G)** Analysis of naive (CD62L+CD45RO–), central memory (CD62L+CD45RO+), effector memory (CD62L-CD45RO+) and terminal effector (CD62L-CD45RO-) cells of CAR-CIK and CAR-T treated animals retrieved from the indicated organs. Mean ± SEM of n=3–6. **(H)** Representative immunohistochemistry images from liver, lungs and spleen of animals treated with CAR-CIK or CAR-T cells or controls, showing hCD45+ effector cells (pink-red) among tumor cells (green) and parenchyma (all cell nuclei in blue). 20-fold magnification, scale bar indicates 100 µM. Group differences with p < 0.05 (*), p < 0.01 (**) were considered statistically significant.

Blood, bone marrow (BM), spleen, liver and lungs were collected from each animal at the time of death to assess persistence and distribution of effector cells. Human CD45+ (hCD45+) cells were detectable via flow cytometry in all assessed organs of 6/10 CAR-CIK-treated animals (not being detected in any organ in 2/10 animals and detected in BM and lungs of n=1 and only in the blood of n=1; **Figure 6A**, **Supplementary Figure 3**). In contrast, only 2/12 CAR-T-treated animals had detectable hCD45+ cells in all assessed organs (not detected in any organ in 2/12 animals and detected in multiple but not all organs in 8/12; **Figure 6A**, **Supplementary Figure 3**). Among hCD45+ cells, we observed equal proportions of GFP+ CAR-bearing cells among CAR-CIK- and CAR-T-treated animals (**Figure 6B**). In blood, BM, spleen and liver samples, the majority of analyzed cells from CAR-CIK-treated animals were CD8+, whereas cells from CAR-T-treated animals were mostly CD8- (**Figures 6C, D**). CAR-CIK and CIK cells retrieved from the assessed organs retained their composition of a mixed TNK cell phenotype (**Figures 6E, F**), whereas CAR-T cells had a sole CD3+ T cell phenotype (**Supplementary Figure 3D**). In all analyzed organs from animals treated with both CAR-CIK and CAR-T cells, the majority of retrieved cells exhibited an effector memory phenotype, with smaller populations of central memory and late effector cells (**Figure 6G**). Finally, immunohistochemistry analysis for hCD45+ cells in representative organ samples confirmed migration of both CAR-effector cells to lungs, livers and spleens (**Figure 6H**). Despite the constraints of these endpoint analyses, these findings show that CD8+ effector phenotype CAR-CIK cells migrated to multiple organs affected by metastatic RMS and indicate effector cell persistence for an extended period *in vivo*.

## 4 Discussion

The advance of CAR technology has revolutionized the treatment of certain hematological malignancies, achieving very promising results, both in a clinical-trial context and in the real-world (1–5). However, the success of CAR-T cell therapy is still limited for solid tumors (6, 7). Several factors impede CAR-T cell applications, such as insufficient homing into the tumor-afflicted organs, tumor heterogeneity, antigen escape and the hypoxic and immunosuppressive environment in bulk tumors. Tumor-associated regulatory T cells and macrophages, as well as ligands for checkpoint receptors expressed by the tumor cells suppress effector cell function *in situ* (7–9). Further, heavy pretreatment of metastatic tumor patients compromises the number and quality of autologous T cells, leading to dysfunction, exhaustion and poor persistence (6, 10).

One strategy to improve adoptive cellular therapy in solid cancer is the use of healthy donor cells for CAR-engineering (9, 10, 12, 13). Allogeneic T cells cannot be used as effector cells outside the setting of allo-HSCT due to potentially lethal GvHD without additional genetic modifications that add complexity to the manufacturing process and introduce new safety concerns (10, 12). By contrast, CIK cells are a population of potent effector cells that can be safely employed across allogeneic barriers (16–20). We and others have previously shown that RMS can be successfully targeted using ErbB2-specific CAR-CIK cells (34, 41, 43, 44). We chose RMS as a model to benchmark CAR-CIK cells against conventional CAR-T cells because there is a high clinical need to develop new treatment strategies for pediatric metastatic RMS (39). To our knowledge, this is the first study to directly compare the efficacy and mode of action of these two immune effectors generated from same-donor samples (52).

To facilitate clinical translation, we utilized a second-generation CD28-CD3ζ CAR previously shown to mediate ErbB2-specific effects (34, 47, 53). A very similar CD28-CD3ζ CAR harboring the same FRP5-derived single-chain fragment variable antibody domain has been safely employed in phase I/II CAR-T clinical trials in pediatric patients with ErbB2+ sarcoma (41–43). In our study, both T and CIK cells from several independent donors could easily be transduced with lentiviral vector particles and displayed robust CAR expression. We confirmed that the typical CD3+CD56+ TNK cell phenotype of CIK cells also develops after lentiviral transduction (34, 44). By optimizing transduction methods, we ensured equal CAR-expression among the bulk populations of CAR-CIK and CAR-T cells used in all experiments. CAR-CIK cells consistently showed more effective killing of RMS cell lines and primary patient tumor cells in 2D culture and inhibited tumor spheroid growth more potently than CAR-T cells, albeit not statistically significant across all E:T ratios. Importantly, only CAR-CIK cells expanded and changed their phenotype upon RMS tumor target exposure. Despite being a predominant T cell population, CAR-CIK cells apparently conveyed their cytotoxicity via NK cell mechanisms of action. These results underline that both effector cell populations differ not only in their phenotype, but also with regard to their functionality. Finally, a xenograft model of metastatic RMS showed equal potency and improved persistence of CAR-CIK cells *in vivo*.

CIK cells have a much lower allo-reactive potential than T cells and maintain their low allo-reactivity after CAR-engineering (16–20). They can be safely applied to patients at dosages more than ten times exceeding the usual T-cell dosage for donor lymphocyte infusions in a haploidentical setting after HSCT (refs (20, 54). and unpublished results of the FFM-CIK-Cell Study 01, Eudra-CT: 2013-005446-11). Allogeneic CIK cells have been approved in Germany as an advanced therapy medicinal product (ATMP) for children and adults with impending relapse of hematologic malignancies after allo-HSCT (ATMP §4b Abs. 3 AMG, license number: PEI.A.11630.01.1) (55). In pediatric RMS patients, one envisioned avenue of translation would be to use matched sibling or parent donors to ensure ample and healthy starting material and reduce any residual allo-reactive potential. Unlike CAR-T cells, allogeneic CAR-CIK cells can be used without additional genetic engineering of the original T cell receptor, thus improving *in vivo* persistence (56) and avoiding additional costs and potential genotoxicity (10, 12).

In the clinic, cytokine-release syndrome (CRS) and neurotoxicity represent the most relevant and potentially life-threatening side effects of CAR-T cell therapy (57). Their pathophysiology has not been fully understood and it is difficult to predict outcomes based on immunodeficient *in vivo* models (57). However, all clinical studies involving CIK and CAR-CIK cells have consistently shown excellent safety profiles (15, 21). Furthermore, encouraging complete response rates without any GvHD or neurotoxicity (and only grade 1–2 CRS) were recently reported in relapsed B-ALL patients after allo-HSCT using donor-derived anti-CD19-CAR-CIK cells (NCT03389035) (18). In our own analyses, CAR-CIK cell cytokine signature showed significantly higher levels of effector molecules such as INFγ, perforin and granulysin. By contrast, CAR-T cells showed high levels of IL-2 and IL-10 which have been linked to CRS (57) and also exhibited increased levels of cytokines linked to immunosuppression, such as IL-4 and IL-10 (58). In our phenotypical characterizations, we showed that CAR-CIK cells have a TNK cell phenotype and whole cell proteomics further indicated that their cytotoxicity is mediated via NK cell mode of actions. When used as adoptive cellular therapies, NK cells have so far not shown side effects of CAR-T cell therapy and demonstrated a favorable safety profile (59). Although a direct clinical safety evaluation is beyond the scope of this work, these results, together with the established safety record of CIK and NK cell therapies, support the clinical advancement of CAR-CIK cells.

Using CIK cells as effector cells for CAR-engineering allows to combine CAR-mediated target specificity of synthetic biology with the potency of innate effectors. Loss of target antigen and antigen heterogeneity have emerged as major obstacles to single-target CAR-immunotherapy (6, 8, 9). As a heterogenous population, CIK cells can recognize and target tumor cells in an HLA-dependent as well as an HLA-independent manner (15, 36, 37). We have previously shown that ErbB2-CAR-CIK cells convey potent cytotoxicity against various RMS cell lines *in vitro* while retaining NKG2D-mediated cytotoxicity against ErbB2-negative tumors (34). Furthermore, CAR-mediated activation of CAR-CIK cells can improve their HLA-independent recognition of target cells (52) and support the adoptive immune system as well as bystander cells within the TME (44). Previous studies have shown that the cytotoxicity of (CAR-)CIK cells is conveyed by their mixed heterogenous population (35) and multiple clinical trials have established the use of CIK cells as composite effector cells (21). We therefore used the bulk population of effector cells for our comparative analyses, encompassing CAR-transduced cells as well as NK an TNK cells, in which viral transduction rates were lower.

Elevated secretion of INFγ seems to be a key feature in CAR-CIK cell mediated cytotoxicity. It is not only important in promoting their reduced GvHD-potential (16), but also required for CAR-cell-mediated killing in solid tumors (60). INFγ signaling facilitates invasion of CAR-cells into tumor islets (61) and may promote the upregulation of MHC molecules on tumor targets and thereby enhance HLA-dependent toxicity (62). INFγ also activates macrophages and antigen-presenting cells, which can recruit and enhance the endogenous antitumor immune responses in an immunocompetent host (63). Although there is evidence for the role of INFγ in creating an immunosuppressive TME (63), we observed no increase in regulatory T cells among CAR-CIK cells and measured decreased expression of the immune checkpoint receptor/exhaustion marker PD-1 on CAR-CIK cells despite similar activation and expansion of both CAR-CIK and CAR-T cells after *in vitro* culture (as indicated by their CD69 expression; **Supplementary Figure 1**). We observed that CD39 was preferentially expressed in CAR-CIK cells, a molecule known to modulate immune responses through adenosine production (64). While CD39 is associated with exhaustion in T cells (64–66), its role in NK cells and the heterogeneous TNK population of CAR-CIK cells remains inconclusive (67–69). Emerging evidence suggests that CD39 in NK cells may support activation and is linked to IL-15 stimulation (68). Importantly, the only other significantly differentially expressed marker commonly linked to exhaustion in our analysis was CTLA4 (70), which was increased in CAR-T cells. Based on these findings and our flow cytometric analyses, we cannot claim that either CAR-CIK or CAR-T cells are more prone to exhaustion. Further investigation into the mechanisms of exhaustion and persistence in these effector cells would provide important insights and represents a promising direction for future research.

The efficacy of CAR-immunotherapy is highly dependent upon prior reduction of tumor load (4) and especially in the context of solid tumors CAR-effector cells will most likely not induce a remission in bulky disease (6). Hence, we used an established *in vivo* model with minimal residual tumor burden at the time of CAR cell infusion (35, 44, 46, 49), which develops into metastasized RMS if left untreated and is similar to other sarcoma mouse models used for immunotherapy (71, 72). Both CAR-effector cells mediated strong inhibition of tumor growth. The *in vivo* trafficking and biodistribution of CAR-CIK and CAR-T cells was analyzed by flow cytometry as well as IHC. Although the predominant effector memory phenotype of CIK cells has previously been associated with reduced persistence (27), we observed effector cells in all assessed organs of the majority of CAR-CIK-treated animals, compared to only a minority of CAR-T-treated animals, up to 100 days after a single injection. This may indicate sustained functionality and vitality of CAR-CIK cells and their ability to maintain efficacy over an extended period *in vivo*. Further analyses of these effector cells retrieved from target organs at multiple pre-planned time points after injection, including detailed phenotypic characterization and assessment of their *ex vivo* functionality, would provide valuable insights into differences in their modes of action and failure. Additionally, investigating the role of exhaustion in these effector cells would be highly informative and could elucidate mechanisms underlying their long-term *in vivo* persistence and efficacy. Clinically, we see a role for CAR-immunotherapy in RMS not against bulky disease, but as a sequenced treatment to control residual disease after surgery and intensive chemo- and radiotherapy. As demonstrated in the clinical HER2-CAR-T trial by Hegde et al. repeated infusions of CAR-effector cells are probably necessary to achieve long lasting remission (43).

The conclusions of our work are limited to the conditions that we have analyzed. Although we have made use of multiple donors and different target cells, including primary patient material, we have only employed one CAR-design and cannot currently generalize beyond rhabdomyosarcoma. We have primarily focused our analyses on the efficacy of these effector cells, their cytokine responses, and the distinct phenotypic and functional properties associated with their mixed T cell and NK cell characteristics. While an in-depth comparison of factors linked to exhaustion and persistence would be highly valuable, it was beyond the scope of the current study. Furthermore, it is necessary to acknowledge the limitations of an immunodeficient mouse model, particularly regarding the absence of the original tumor microenvironment observed in patients and lack of additional immunosuppressive cells (73). However, immunodeficient mouse models are commonly used to assess efficacy (6, 71, 72) and this model allowed us to utilize the established ErbB2-specific CD28-CD3ζ CAR (34, 47, 53) and human effector cells to facilitate clinical translation.

Taken together, this first comparison of CAR-CIK vs. CAR-T cells in solid tumors demonstrates that ErbB2-CAR cells generated from CIK cells can be robustly produced by lentiviral transduction and have more potent *in vitro* activity and equally strong *in vivo* potency as CAR-T cells. CAR-CIK cells exhibit phenotypic differences and distinct modes of actions characterized by a CD3+CD56+ TNK and effector phenotype and rely on NK cell modes of action, while demonstrating enhanced persistence compared to adoptively transferred NK cells (74). Due to their intrinsically varied endogenous antigen-specific and non-antigen-specific anti-tumor potency, they can provide additional benefits to the CAR. Importantly, their use as healthy donor cells in an allogeneic setting is feasible and safe without additional multi-level genetic engineering. These findings support the development of CAR-CIK cells as allogeneic immune effectors to advance cellular therapies in metastatic RMS and other solid tumors.

## Data Availability

MS-data are available on the public open-access repository PRIDE (https://www.ebi.ac.uk/pride/; dataset identifier PXD050654). Please direct any inquiries or requests for further raw data or additional information to the corresponding authors.

## References

[1] Maude SL Frey N Shaw PA Aplenc R Barrett DM Bunin NJ. Chimeric antigen receptor T cells for sustained remissions in leukemia. N Engl J Med. (2014) 371:1507–17. doi: 10.1056/NEJMoa1407222 PMC4267531 25317870

[2] Gardner RA Shah NN. CAR T-cells for cure in pediatric B-ALL. J Clin Oncol. (2023) 41:1646–8. doi: 10.1200/JCO.22.02345 PMC10043577 36634289

[3] Myers RM Jacoby E Pulsipher MA Pasquini MC Grupp SA Shah NN. INSPIRED Symposium Part 1: Clinical Variables Associated with Improved Outcomes for Children and Young Adults treated with Chimeric Antigen Receptor T cells for B cell Acute Lymphoblastic Leukemia. Transplant Cell Ther. (2023) 29:598–607. doi: 10.1016/j.jtct.2023.07.016 37481241 PMC11031134

[4] Cappell KM Kochenderfer JN. Long-term outcomes following CAR T cell therapy: what we know so far. Nat Rev Clin Oncol. (2023) 20:359–71. doi: 10.1038/s41571-023-00754-1 PMC10100620 37055515

[5] Maude SL Laetsch TW Buechner J Rives S Boyer M Bittencourt H. Tisagenlecleucel in children and young adults with B-cell lymphoblastic leukemia. N Engl J Med. (2018) 378:439–48. doi: 10.1056/NEJMoa1709866 PMC5996391 29385370

[6] Albelda SM. CAR T cell therapy for patients with solid tumours: key lessons to learn and unlearn. Nat Rev Clin Oncol. (2024) 21:47–66. doi: 10.1038/s41571-023-00832-4 37904019

[7] Hou AJ Chen LC Chen YY. Navigating CAR-T cells through the solid-tumour microenvironment. Nat Rev Drug Discov. (2021) 20:531–50. doi: 10.1038/s41573-021-00189-2 33972771

[8] Rafiq S Hackett CS Brentjens RJ. Engineering strategies to overcome the current roadblocks in CAR T cell therapy. Nat Rev Clin Oncol. (2020) 17:147–67. doi: 10.1038/s41571-019-0297-y PMC7223338 31848460

[9] Labanieh L Mackall CL. CAR immune cells: design principles, resistance and the next generation. Nature. (2023) 614:635–48. doi: 10.1038/s41586-023-05707-3 36813894

[10] Depil S Duchateau P Grupp SA Mufti G Poirot L. Off-the-shelf’ allogeneic CAR T cells: development and challenges. Nat Rev Drug Discov. (2020) 19:185–99. doi: 10.1038/s41573-019-0051-2 31900462

[11] Elavia N Panch SR McManus A Bikkani T Szymanski J Highfill SL. Effects of starting cellular material composition on chimeric antigen receptor T-cell expansion and characteristics. Transfusion (Paris). (2019) 59:1755–64. doi: 10.1111/trf.15287 30973976

[12] Themeli M Rivière I Sadelain M. New cell sources for T cell engineering and adoptive immunotherapy. Cell Stem Cell. (2015) 16:357–66. doi: 10.1016/j.stem.2015.03.011 PMC5611846 25842976

[13] Hossian AKMN Hackett CS Brentjens RJ Rafiq S. Multipurposing CARs: Same engine, different vehicles. Mol Ther. (2022) 30:1381–95. doi: 10.1016/j.ymthe.2022.02.012 PMC9077369 35151842

[14] Wu X Schmidt-Wolf IGH. An alternative source for allogeneic CAR T cells with a high safety profile. Front Immunol. (2022) 13:1–4. doi: 10.3389/fimmu.2022.913123 PMC9170073 35677035

[15] Zhang Y Schmidt-Wolf IGH. Ten-year update of the international registry on cytokine-induced killer cells in cancer immunotherapy. J Cell Physiol. (2020) 235:9291–303. doi: 10.1002/jcp.v235.12 32484595

[16] Baker J Verneris MR Ito M Shizuru JA Negrin RS. Expansion of cytolytic CD8+ natural killer T cells with limited capacity for graft-versus-host disease induction due to interferon γ production. Blood. (2001) 97:2923–31. doi: 10.1182/blood.V97.10.2923 11342413

[17] Nishimura R Baker J Beilhack A Zeiser R Olson JA Sega EI. *In vivo* trafficking and survival of cytokine-induced killer cells resulting in minimal GVHD with retention of antitumor activity. Blood. (2008) 112:2563–74. doi: 10.1182/blood-2007-06-092817 PMC2532819 18565854

[18] Magnani CF Gaipa G Lussana F Belotti D Gritti G Napolitano S. Sleeping Beauty–engineered CAR T cells achieve antileukemic activity without severe toxicities. J Clin Invest. (2020) 130:6021–33. doi: 10.1172/JCI138473 PMC7598053 32780725

[19] Oelsner S Wagner J Friede ME Pfirrmann V Genßler S Rettinger E. Chimeric antigen receptor-engineered cytokine-induced killer cells overcome treatment resistance of pre-B-cell acute lymphoblastic leukemia and enhance survival. Int J Cancer. (2016) 139:1799–809. doi: 10.1002/ijc.v139.8 27253354

[20] Merker M Salzmann-Manrique E Katzki V Huenecke S Bremm M Bakhtiar S. Clearance of hematologic Malignancies by allogeneic cytokine-induced killer cell or donor lymphocyte infusions. Biol Blood Marrow Transplant. (2019) 25:1281–92. doi: 10.1016/j.bbmt.2019.03.004 30878607

[21] Sharma A Ren X Rosato A Sangiolo D Wang Z Tettamanti S. Cytokine-induced killer (CIK) cells, successes and challenges: report on the first international conference dedicated to the clinical translation of this unique adoptive cell immunotherapy. Cancer Immunol Immunother. (2024) 73:21. doi: 10.1007/s00262-023-03605-1 38279995 PMC10821962

[22] Bendelac A Lantz O Quimby ME Yewdell JW Bennink JR Brutkiewicz RR. CD1 recognition by mouse NK1+ T lymphocytes. Science. (1995) 268:863–5. doi: 10.1126/science.7538697 7538697

[23] Bendelac A Rivera MN Park SH Roark JH. Mouse CD1-specific NK1 T cells: development, specificity, and function. Annu Rev Immunol. (1997) 15:535–62. doi: 10.1146/annurev.immunol.15.1.535 9143699

[24] Imai K Kanno M Kimoto H Shigemoto K Yamamoto S Taniguchi M. Sequence and expression of transcripts of the T-cell antigen receptor alpha-chain gene in a functional, antigen-specific suppressor-T-cell hybridoma. Proc Natl Acad Sci U S A. (1986) 83:8708–12. doi: 10.1073/pnas.83.22.8708 PMC387000 2946043

[25] Shin S El-Diwany R Schaffert S Adams EJ Garcia KC Pereira P. Antigen recognition determinants of γδ T cell receptors. Science. (2005) 308:252–5. doi: 10.1126/science.1106480 15821090

[26] Nörenberg J Jaksó P Barakonyi A. Gamma/delta T cells in the course of healthy human pregnancy: cytotoxic potential and the tendency of CD8 expression make CD56+ γδT cells a unique lymphocyte subset. Front Immunol. (2021) 11:596489. doi: 10.3389/fimmu.2020.596489 33603738 PMC7884463

[27] Franceschetti M Pievani A Borleri G Vago L Fleischhauer K Golay J. Cytokine-induced killer cells are terminallydifferentiated activated CD8 cytotoxic T-EMRA lymphocytes. Exp Hematol. (2009) 37:616–28. doi: 10.1016/j.exphem.2009.01.010 19375652

[28] Gütgemann S Frank S Strehl J Schmidt-Wolf IGH. Cytokine-induced killer cells are type II natural killer T cells. GMS Ger Med Sci. (2007) 5:Doc07. 19675715 PMC2703238

[29] Cappuzzello E Sommaggio R Zanovello P Rosato A. Cytokines for the induction of antitumor effectors: The paradigm of Cytokine-Induced Killer (CIK) cells. Cytokine Growth Factor Rev. (2017) 36:99–105. doi: 10.1016/j.cytogfr.2017.06.003 28629761

[30] Verneris MR Baker J Edinger M Negrin RS. Studies of ex vivo activated and expanded CD8+ NK-T cells in humans and mice. J Clin Immunol. (2002) 22:131–6. doi: 10.1023/A:1015415928521 12078854

[31] Joshi PS Liu JQ Wang Y Chang X Richards J Assarsson E. Cytokine-induced killer T cells kill immature dendritic cells by TCR-independent and perforin-dependent mechanisms. J Leukoc Biol. (2006) 80:1345–53. doi: 10.1189/jlb.0506305 16997856

[32] Liang G Feng G Chen H Li L Hu J Zhou C. Changes in the TCR repertoire of T-cell subsets during culture of cytokine-induced killer cells. FEBS Lett. (2022) 596:2696–705. doi: 10.1002/1873-3468.14501 36129621

[33] Ortaldo JR Winkler-Pickett RT Yagita H Young HA. Comparative studies of CD3- and CD3+ CD56+ cells: examination of morphology, functions, T cell receptor rearrangement, and pore-forming protein expression. Cell Immunol. (1991) 136:486–95. doi: 10.1016/0008-8749(91)90369-M 1714795

[34] Merker M Pfirrmann V Oelsner S Fulda S Klingebiel T Wels WS. Generation and characterization of ErbB2-CAR-engineered cytokine-induced killer cells for the treatment of high-risk soft tissue sarcoma in children. Oncotarget. (2017) 8:66137–53. doi: 10.18632/oncotarget.19821 PMC5630399 29029499

[35] Rettinger E Kreyenberg H Merker M Kuçi S Willasch A Bug G. Immunomagnetic selection or irradiation eliminates alloreactive cells but also reduces anti-tumor potential of cytokine-induced killer cells: implications for unmanipulated cytokine-induced killer cell infusion. Cytotherapy. (2014) 16:835–44. doi: 10.1016/j.jcyt.2014.01.003 24582456

[36] Schmidt-Wolf IGH Negrin RS Kiem HP Blume KG Weissman IL. Use of a SCID mouse/human lymphoma model to evaluate cytokine-induced killer cells with potent antitumor cell activity. J Exp Med. (1991) 174:139–49. doi: 10.1084/jem.174.1.139 PMC2118875 1711560

[37] Pievani A Borleri G Pende D Moretta L Rambaldi A Golay J. Dual-functional capability of CD3+CD56+ CIK cells, a T-cell subset that acquires NK function and retains TCR-mediated specific cytotoxicity. Blood. (2011) 118:3301–10. doi: 10.1182/blood-2011-02-336321 21821703

[38] Yoon SH Lee JM Woo SJ Park MJ Park JS Kim HS. Transfer of her-2/neu specificity into cytokine-induced killer (CIK) cells with RNA encoding chimeric immune receptor (CIR). J Clin Immunol. (2009) 29:806–14. doi: 10.1007/s10875-009-9308-6 19517218

[39] Skapek SX Ferrari A Gupta AA Lupo PJ Butler E Shipley J. Rhabdomyosarcoma. Nat Rev Dis Primer. (2019) 5:41572. doi: 10.1038/s41572-018-0051-2 PMC7456566 30617281

[40] De Giovanni C Landuzzi L Palladini A Nicoletti G Nanni P Lollini PL. HER tyrosine kinase family and rhabdomyosarcoma: role in onset and targeted therapy. Cells. (2021) 10:1808. doi: 10.3390/cells10071808 34359977 PMC8305095

[41] Ahmed N Brawley VS Hegde M Robertson C Ghazi A Gerken C. Human epidermal growth factor receptor 2 (HER2) - Specific chimeric antigen receptor - Modified T cells for the immunotherapy of HER2-positive sarcoma. J Clin Oncol. (2015) 33:1688–96. doi: 10.1200/JCO.2014.58.0225 PMC4429176 25800760

[42] Hegde M Joseph SK Pashankar F DeRenzo C Sanber K Navai S. Tumor response and endogenous immune reactivity after administration of HER2 CAR T cells in a child with metastatic rhabdomyosarcoma. Nat Commun. (2020) 11:3549. doi: 10.1038/s41467-020-17175-8 32669548 PMC7363864

[43] Hegde M Navai S DeRenzo C Joseph SK Sanber K Wu M. Autologous HER2-specific CAR T cells after lymphodepletion for advanced sarcoma: a phase 1 trial. Nat Cancer. (2024) 5:880–94. doi: 10.1038/s43018-024-00749-6 PMC11588040 38658775

[44] Merker M Wagner J Kreyenberg H Heim C Moser LM Wels WS. ERBB2-CAR-engineered cytokine-induced killer cells exhibit both CAR-mediated and innate immunity against high-risk rhabdomyosarcoma. Front Immunol. (2020) 11:1–13. doi: 10.3389/fimmu.2020.581468 33193388 PMC7641627

[45] Abel T El Filali E Waern J Schneider IC Yuan Q Münch RC. Specific gene delivery to liver sinusoidal and artery endothelial cells. Blood. (2013) 122:2030–8. doi: 10.1182/blood-2012-11-468579 23884859

[46] Rettinger E Meyer V Kreyenberg H Volk A Kuçi S Willasch A. Cytotoxic capacity of IL-15-stimulated cytokine-induced killer cells against human acute myeloid leukemia and rhabdomyosarcoma in humanized preclinical mouse models. Front Oncol. (2012) 2:1–12. doi: 10.3389/fonc.2012.00032 PMC3356002 22655268

[47] Schönfeld K Sahm C Zhang C Naundorf S Brendel C Odendahl M. Selective inhibition of tumor growth by clonal NK cells expressing an erbB2/HER2-specific chimeric antigen receptor. Mol Ther. (2015) 23:330–8. doi: 10.1038/mt.2014.219 PMC4445620 25373520

[48] Demaison C Parsley K Brouns G Scherr M Battmer K Kinnon C. High-level transduction and gene expression in hematopoietic repopulating cells using a human imunodeficiency virus type 1-based lentiviral vector containing an internal spleen focus forming virus promoter. Hum Gene Ther. (2002) 13:803–13. doi: 10.1089/10430340252898984 11975847

[49] Heim C Moser LM Kreyenberg H Bonig HB Tonn T Wels WS. ErbB2 (HER2)-CAR-NK-92 cells for enhanced immunotherapy of metastatic fusion-driven alveolar rhabdomyosarcoma. Front Immunol. (2023) 14:1228894. doi: 10.3389/fimmu.2023.1228894 37662907 PMC10471977

[50] Perez-Riverol Y Csordas A Bai J Bernal-Llinares M Hewapathirana S Kundu DJ. The PRIDE database and related tools and resources in 2019: improving support for quantification data. Nucleic Acids Res. (2019) 47:D442–50. doi: 10.1093/nar/gky1106 PMC6323896 30395289

[51] Segovis CM Schoon RA Dick CJ Nacusi LP Leibson PJ Billadeau DD. PI3K links NKG2D signaling to a crkL pathway involved in natural killer cell adhesion, polarity, and granule secretion. J Immunol. (2009) 182:6933–42. doi: 10.4049/jimmunol.0803840 PMC2706535 19454690

[52] Hombach AA Rappl G Abken H. Arming cytokine-induced killer cells with chimeric antigen receptors: CD28 outperforms combined CD28–OX40 “Super-stimulation. Mol Ther. (2013) 21:2268–77. doi: 10.1038/mt.2013.192 PMC3863798 23985696

[53] Burger MC Forster MT Romanski A Straßheimer F Macas J Zeiner PS. Intracranial injection of natural killer cells engineered with a HER2-targeted chimeric antigen receptor in patients with recurrent glioblastoma. Neuro-Oncol. (2023) 25:2058–71. doi: 10.1093/neuonc/noad087 PMC10628939 37148198

[54] Rettinger E Huenecke S Bonig H Merker M Jarisch A Soerensen J. Interleukin-15-activated cytokine-induced killer cells may sustain remission in leukemia patients after allogeneic stem cell transplantation: feasibility, safety and first insights on efficacy. Haematologica. (2016) 101:e153–6. doi: 10.3324/haematol.2015.138016 PMC5004389 26768688

[55] Bremm M Pfeffermann LM Cappel C Katzki V Erben S Betz S. Improving clinical manufacturing of IL-15 activated cytokine-induced killer (CIK) cells. Front Immunol. (2019) 10:84–94. doi: 10.3389/fimmu.2019.01218 31214182 PMC6554420

[56] Stenger D Stief TA Kaeuferle T Willier S Rataj F Schober K. Endogenous TCR promotes *in vivo* persistence of CD19-CAR-T cells compared to a CRISPR/Cas9-mediated TCR knockout CAR. Blood. (2020) 136:1407–18. doi: 10.1182/blood.2020005185 PMC7612202 32483603

[57] Morris EC Neelapu SS Giavridis T Sadelain M. Cytokine release syndrome and associated neurotoxicity in cancer immunotherapy. Nat Rev Immunol. (2022) 22:85–96. doi: 10.1038/s41577-021-00547-6 34002066 PMC8127450

[58] Bell M Gottschalk S. Engineered cytokine signaling to improve CAR T cell effector function. Front Immunol. (2021) 12:1–16. doi: 10.3389/fimmu.2021.684642 PMC8220823 34177932

[59] Laskowski TJ Biederstädt A Rezvani K. Natural killer cells in antitumour adoptive cell immunotherapy. Nat Rev Cancer. (2022) 22:557–75. doi: 10.1038/s41568-022-00491-0 PMC9309992 35879429

[60] Larson RC Kann MC Bailey SR Haradhvala NJ Llopis PM Bouffard AA. CAR T cell killing requires the IFNγR pathway in solid but not liquid tumours. Nature. (2022) 604:563–70. doi: 10.1038/s41586-022-04585-5 35418687

[61] Kantari-Mimoun C Barrin S Vimeux L Haghiri S Gervais C Joaquina S. CAR T-cell entry into tumor islets is a two-step process dependent on IFNγ and ICAM-1. Cancer Immunol Res. (2021) 9:1425–38. doi: 10.1158/2326-6066.CIR-20-0837 34686489

[62] van den Elsen PJ. Expression regulation of major histocompatibility complex class I and class II encoding genes. Front Immunol. (2011) 2:1–9. doi: 10.3389/fimmu.2011.00048 22566838 PMC3342053

[63] Alspach E Lussier DM Schreiber RD. Interferon γ and its important roles in promoting and inhibiting spontaneous and therapeutic cancer immunity. Cold Spring Harb Perspect Biol. (2019) 11:a028480. doi: 10.1101/cshperspect.a028480 29661791 PMC6396335

[64] Moesta AK Li XY Smyth MJ. Targeting CD39 in cancer. Nat Rev Immunol. (2020) 20:739–55. doi: 10.1038/s41577-020-0376-4 32728220

[65] Simoni Y Becht E Fehlings M Loh CY Koo SL Teng KWW. Bystander CD8+ T cells are abundant and phenotypically distinct in human tumour infiltrates. Nature. (2018) 557:575–9. doi: 10.1038/s41586-018-0130-2 29769722

[66] Canale FP Ramello MC Núñez N Araujo Furlan CL Bossio SN Gorosito Serrán M. CD39 expression defines cell exhaustion in tumor-infiltrating CD8+ T cells. Cancer Res. (2018) 78:115–28. doi: 10.1158/0008-5472.CAN-16-2684 29066514

[67] Duhen T Duhen R Montler R Moses J Moudgil T de Miranda NF. Co-expression of CD39 and CD103 identifies tumor-reactive CD8 T cells in human solid tumors. Nat Commun. (2018) 9:2724. doi: 10.1038/s41467-018-05072-0 30006565 PMC6045647

[68] Kang G Zhao X Sun J Cheng C Wang C Tao L. A2AR limits IL-15-induced generation of CD39+ NK cells with high cytotoxicity. Int Immunopharmacol. (2023) 114:109567. doi: 10.1016/j.intimp.2022.109567 36529024

[69] Yan J Li XY Roman Aguilera A Xiao C Jacoberger-Foissac C Nowlan B. Control of metastases via myeloid CD39 and NK cell effector function. Cancer Immunol Res. (2020) 8:356–67. doi: 10.1158/2326-6066.CIR-19-0749 31992567

[70] Walker LSK Sansom DM. The emerging role of CTLA4 as a cell-extrinsic regulator of T cell responses. Nat Rev Immunol. (2011) 11:852–63. doi: 10.1038/nri3108 22116087

[71] Tian M Wei JS Shivaprasad N Highfill SL Gryder BE Milewski D. Preclinical development of a chimeric antigen receptor T cell therapy targeting FGFR4 in rhabdomyosarcoma. Cell Rep Med. (2023) 4:101212. doi: 10.1016/j.xcrm.2023.101212 37774704 PMC10591056

[72] Ahmed N Salsman VS Yvon E Louis CU Perlaky L Wels WS. Immunotherapy for osteosarcoma: genetic modification of T cells overcomes low levels of tumor antigen expression. Mol Ther. (2009) 17:1779–87. doi: 10.1038/mt.2009.133 PMC2835000 19532139

[73] Duncan BB Dunbar CE Ishii K. Applying a clinical lens to animal models of CAR-T cell therapies. Mol Ther - Methods Clin Dev. (2022) 27:17–31. doi: 10.1016/j.omtm.2022.08.008 36156878 PMC9478925

[74] Page A Chuvin N Valladeau-Guilemond J Depil S. Development of NK cell-based cancer immunotherapies through receptor engineering. Cell Mol Immunol. (2024) 21:315–31. doi: 10.1038/s41423-024-01145-x PMC10978891 38443448

