# Supplementary material for: CAR-CIK vs. CAR-T: benchmarking novel cytokine-induced killer cells as solid tumor immunotherapy in ErbB2+ rhabdomyosarcoma
Source: Front Immunol. 2025 Feb 3;16:1485817. doi: 10.3389/fimmu.2025.1485817 (PMC11831232; doi:10.3389/fimmu.2025.1485817)
Supplement: Supplementary file 1 [file DataSheet1.docx]

Supplementary Material

# Supplementary Methods

**Cell lines**

The alveolar RMS cell lines Rh30 (PAX3-FOXO1/FKHR fusion-positive) and Rh41 (PAX3-FOXO1/FKHR fusion-positive, p53 deletion mutation) were initially purchased from DSMZ (Deutsche Sammlung von Mikroorganismen und Zellkulturen GmbH). Cells were cultured in Roswell Park Memorial Institute (RPMI) 1640 medium with GlutaMAX (ThermoFisher) and 10 % fetal bovine serum (FBS; Sigma-Aldrich). FBS was heat-inactivated for all applications. HEK293T/17 cells were initially obtained from ATCC (CRL-11268) and cultured in DMEMHighGlucose (ThermoFisher) with 10 % FBS. Regular mycoplasm testing was carried out via PCR (1). Cell lines were used for experiments within five passages or less of being thawed from frozen stocks. All cell lines were cultured in a humidified 5 % CO_2_ atmosphere at 37 °C.

**Lentiviral particles and transduction of cell lines**

Lentiviral vector particles were produced by transfecting HEK293T/17 cells with the respective transfer plasmid together with packaging and envelope plasmids pCMVdR8.91 (2) and pMD2.G (Addgene #12259) by polyethylenimine (Sigma-Aldrich) transfection. GFP/fLuciferase-expressing cells were generated by lentiviral transduction using polybrene with the pSIEW-luc2 plasmid encoding enhanced green fluorescent protein (eGFP) and firefly luciferase linked by a T2A peptide, as previously reported (3). mCherry/luciferase-expressing cells were generated by lentiviral transduction using polybrene with the pLenti_fLuc_mCherry plasmid, encoding red fluorescence protein (mCherry) and firefly luciferase linked by a T2A peptide. The pLenti_fLuc_mCherry plasmid was constructed by inserting firefly luciferase (Addgene #24307) into a previously described second-generation lentiviral transfer vector (4) using standard cloning techniques.

**Flow cytometry**

Cells were washed with phosphate-buffered saline (PBS) (ThermoFisher) before staining. Staining was performed in brilliant stain buffer (BD Biosciences) in case of multi-antibody-staining, or PBS for 15 min at 4 °C. All phenotypic characterizations of cultured cells were performed on day 10 of *in vitro* expansion. CAR expression was determined using a chimeric ErbB2-Fc protein (Sino Biological) after Fc-receptor blocking (Human TruStain FcX, BioLegend), followed by staining with an anti-IgG-Fc secondary APC-conjugated antibody (HP6017, BioLegend). Measurements were performed on a FACS Canto 10c device (BD Biosciences). All other measurements were done on a FACSCelesta (BD Biosciences). Data were analyzed using FlowJo software (Version 10.8.2, Tree Star). The following antibodies were used: CD45 PerCP (HI30), CD4 PerCP (OKT4), CD16 Pe (3G8), ErbB2 Pe (24D2), Pe mouse IgG1k isotype control (MOPC-21) and CD45 PacificBlue (HI30) (all from BioLegend); CD19 BV605 (SJ25C1), CD56 BV421 (NCAM16.2), CD3 BUV395 (SK7), CD3 BV786 (SK7), CD45RO BV711 (UCHL1), CD62L BUV737 (SK11), CD8 BUV395 (RPA T8), CD25 BV605 (2A3), CD127 BV421 (HIL-7R-M21), CD69 BUV737 (FN50) and CD56 BUV395 (NCAM 16.2) (all from BD Biosciences).

**Luciferase toxicity assay**

RMS cells stably expressing firefly luciferase (fLuc) were used for 24 h toxicity assays as previously described (5): After allowing targets cells to adhere, effector cells suspended in 100 µl medium were added at E:T ratios from 10:1 to 1.25:1. For maximum target cell lysis, 100 µl of 10 % Triton X-100 solution (Sigma Aldrich) was added. Untreated cells were used as reference. Experiments were set up in triplicates. Cells were co-incubated for 24 h, then 20 µl of a 3 mM luciferin solution (InVivoGlo, Promega) were added per well. After 15 min of incubation at 37 °C, the plate was analyzed using the luminometer function of the GloMAX Multi+ (Promega) instrument with an integration time of 1 s/well. Results are given as percentage of luciferase signal of untreated target cells.

**Whole cell proteomics of effector cells**

For whole cell proteomics CAR-CIK, CAR-T cells and untransduced parental cells were co-incubated ± Rh30 cells at an effector to target ratio of 2:1. After 24h, cells were harvested via aspiration and plates were rinsed with PBS (ThermoFisher) to collect all effector cells. Samples were washed twice with PBS (300 g, 5 min) and were stained for CD45 (CD45PacificBlue (HI30), BioLegend). CD45+ effector cells were isolated via FACS using a BD FACS Aria 3 and collected into 1.5 ml tubes (Eppendorf) containing PBS and spun down (500 g, 5 min) to form a pellet, which was cryo-preserved at -80 °C.

For sample preparation, cells were lysed and denatured with 2 % SDS, 50 mM Tris-HCl pH 8, 150 mM NaCl, 10 mM TCEP, 40 mM chloroacetamide and protease inhibitor (EDTA-free, Roche) at 95 °C for 10 min, then sonicated for 1 min (1 s ON/ 1 s OFF pulse, 45 % amplitude) and boiled again for 5 min at 95 °C. Precipitation of lysates was performed with three volumes of ice-cold methanol, one volume chloroform and 2.5 volumes ddH2O. After centrifugation (18,000 g for 15 min at 4 °C) the upper aqueous phase was aspirated, and three volumes of ice-cold methanol were added. After mixing of samples, proteins were pelleted by centrifugation (18,000 g for 5 min at 4 °C). Protein pellets were washed with ice-cold methanol, dried at room temperature and then resuspended in a solution of 8 M urea, 10 mM EPPS buffer pH 8.2. µBCA assay (ThermoFisher) was used to determine protein concentrations. Per sample, 100 µg of protein were diluted to 1 M urea in 10 mM EPPS pH 8.2 and incubated overnight with LysC (Wako Chemicals,) at 1:50 (w/w) ratio and trypsin (Promega) at a ratio of 1:100 (w/w). Peptides were acidified with trifluoroaceticacid (TFA) to a pH of 2–3 and purified using tC18 SepPak columns (50 mg, Waters) according to the manufacturer’s protocol. After vacuum centrifugation, dried peptides were stored for further processing. Peptides were then resuspended in TMT-labeling buffer (0.2 M EPPS, pH 8.2, 10 % acetonitrile (ACN)) and concentration of peptides was determined by µBCA. TMT reagents (ThermoFisher) were mixed with 20 µg of peptides in 1:2 (w/w) ratio, followed by an incubation at room temperature for 1 hour. Reactions were then quenched by addition of hydroxylamine to a final concentration of 0.5 % (room temperature, 15 min). After a single injection measurement by LC-MS/MS, samples were pooled in equimolar ratios for each channel for offline HPLC (High Performance Liquid Chromatography) fractionation.

Peptides were fractionated using high-pH liquid-chromatography on a micro-flow HPLC (Dionex U3000 RSLC, ThermoFisher). 45 µg of pooled and purified TMT labelled peptides resuspended in Solvent A (5 mM ammonium-bicarbonate, 5 % ACN) were separated on a C18 column (XSelect CSH, 1 mm x 150 mm, 3.5 µm particle size, Waters) using a multistep gradient from 3–60 % Solvent B (5 mM ammonium-bicarbonate, 80 % ACN) over 65 minutes at a flow rate of 30 µl/min. Eluting peptides were collected every 43 seconds from minute 2 for 69 minutes into a total of 96 fractions, which were cross-concatenated into 24 fractions. Pooled fractions were dried in a vacuum concentrator.

For measurement, samples were resuspended in 2 % acetonitrile and 0.1 % formic acid and separated on an Easy nLC 1200 (ThermoFisher) and a 22 cm long, 75 μm ID fused-silica column which had been packed in house with 1.9 μm C18 particles (ReproSil-Pur, Dr. Maisch) and kept at 50 °C using an integrated column oven (Sonation). Assuming equal amounts in each fraction, 500 ng of peptides were eluted by a non-linear gradient from 7 to 40 % ACN over 90 minutes followed by a step-wise increase to 75 % ACN in 6 minutes which was held for another 9 minutes. After that, peptides were directly sprayed into an Orbitrap Fusion Lumos mass spectrometer equipped with a nanoFlex ion source (ThermoFisher). Sprayed peptides were analyzed using the multi-notch MS3-based TMT method in order to minimize ratio compression and ion interference as previously described (6). Full-scan MS spectra (350–1400 m/z) were acquired with a resolution of 120,000 at m/z 200, maximum injection time of 100 ms and AGC (automatic gain control) target value of 4x10^5^. The most intense precursors with a charge state between 2 and 6 per full scan were selected for fragmentation (“Top Speed” with a cycle time of 1.5 seconds) and isolated with a quadrupole isolation window of 0.7 Th. MS2 scans were performed in the Ion trap (Turbo) using a maximum injection time of 50 ms, AGC target value of 1.5x10^4^ and fragmented using collision-induced dissociation (CID) with a normalized collision energy (NCE) of 35 %. Synchronous precursor selection-MS3 (SPS-MS3) scans for quantification were performed on the 10 most intense MS2 fragment ions with an isolation window of 0.7 Th (MS) and 2 m/z (MS2). Ions were fragmented using a higher-energy collisional dissociation (HCD) with an NCE of 65 % and analyzed in the Orbitrap with a resolution of 50,000 at m/z 200, scan range of 100–500 m/z, AGC target value of 1.5x10^5^ and a maximum injection time of 86 ms. Repeated sequencing of already acquired precursors was limited by setting a dynamic exclusion of 60 seconds and 7 ppm and advanced peak determination was deactivated.

Proteomics data analysis:

Raw files were analyzed using Proteome Discoverer (PD) 2.4 (ThermoFisher). Spectra were selected using default settings. For whole cell proteome analysis, database searches for protein identification were performed using SequestHT node in PD against human trypsin digested proteome (Homo sapiens SwissProt database, TaxID:9606, version 12 March 2020). Contaminants were determined using MaxQuant’s “contamination.fasta” for quality control. TMT6 (+229.163) at the N-terminus, TMT6 (K, +229.163) at lysine and carbamidomethyl (+57.021) at cysteine residues were set as fixed modifications. Methionine oxidation (M, +15.995) and acetylation (+42.011) at the protein N-terminus were set as dynamic modifications. Precursor mass tolerance was set to 20 ppm and fragment mass tolerance was set to 0.5 Da. Default percolator settings in PD were used to filter peptide-spectrum matches (PSMs). Only PSMs with a signal-to-noise above 10, a co-isolation below 50 %, as well as at least 50 % SPS matches derived from unique peptides were used for protein quantification after total intensity normalization. The protein file from PD was then exported for further statistical analysis in R. Normalized abundances from the protein file were used for statistical analysis after contaminations and completely empty values were removed.

Filtered data was used as input for a custom-made in-house R pipeline as described previously (4). R version 4.2.2 was used together with data.table 1.14.10. As no relevant differences were observed for the condition ± RMS, results were aggregated for each effector cell. Differential gene expression changes comparing CAR-CIK vs. CAR-T cells were analyzed using DEqMS 1.16.0 (7).

For KEGG pathway analysis, the list of all quantified proteins was ranked (absolute log2 fold chance * -log10-transformed q-value) and gene set enrichment analysis was done with clusterProfiler 4.6.2 using default settings and FDR-correction of p values.

**Detection of immune effector molecules via bead-based immuno-assay**

Supernatants of luciferase toxicity assays were collected after 24 h of co-incubation (E:T 10:1) and stored at -80 °C. Samples were analyzed for their cytokine profile using a bead-based immunoassay (Multi-Analyte Flow Assay Kit LEGENDplex™, Human CS8/NK Panel, 13-plex, BioLegend) following the manufacturer’s instructions. Samples were measured on a FACS Canto 10c device (BD Biosciences) and analyzed using the LEGENDplex data analysis software (BioLegend).

***In vivo* metastatic RMS xenograft model – reporting according to the ARRIVE guidelines**

Animal experiments were approved by and registered in advance with the responsible government oversight committee (Regierungspräsidium Darmstadt, Dezernat V54, ref. FK/2033). All experimental facilities as well as the animal housing were located at the animal facility of Georg-Speyer-Haus in Frankfurt am Main, Germany and complied with Directive 2010/63/EU on the protection of animals used for scientific purposes. Additionally, our practices adhered to the recommendations provided by the Federation of Laboratory Animal Science Associations (FELASA). Reporting was done following the ARRIVE guidelines (8). Female non-obese diabetic (NOD)/severe combined immunodeficient (SCID) /IL-2-receptor-γ−/− (NSG) mice were purchased from Charles-River and used at the age of 6 to 12 weeks. Approximately one week prior to the initiation of the experiments, animals were transferred to their respective cages for acclimatization. To mimic the clinical situation of residual RMS after heavy pretreatment (9) mice were sublethally irradiated with 2.5 Gy (Biobeam 2000) on day -1. One day later (d0), 1x10^5^ Rh30GFP/fLuc+ cells resuspended in 100 μl of PBS were injected via the tail vein, allowing for metastatic tumor spread into liver, lungs and bone marrow. Mice were randomly divided into five different groups: control animals (n=7) received 100 µl medium (GlutaMAX with 10 % FBS) and treatment groups received 2.5x10^6^ effector cells resuspended in 100 µl medium via tail vein injection on day +1. 11 mice received CAR-CIK cells, 12 mice CAR-T cells, 6 mice were treated with parental CIK cells, 5 with parental T cells. The sample size was based on prior experiments. Tumor growth was monitored weekly by bioluminescence imaging (BLI) using an IVIS Lumina II system (Perkin Elmer). Mice were anesthetized via isoflurane inhalation and 1500 μg *in vivo* grade VivoGlo luciferin (Promega) in 100 μl PBS were injected subcutaneously. After 15 min, images were acquired at serial exposure times (1 s–4 min). Moribund mice were sacrificed and peripheral blood, bone marrow (BM), lungs, liver and spleen were isolated and analyzed for persistence of human XQuartz 2.7.11cells as previously described (5): Organs were cut in half and partly conserved in formaldehyde for immunohistochemistry (see below). The remaining organ tissue was processed to single cell suspensions and analyzed via flow cytometry for human CD45+ effector cells.

All applicable guidelines for the housing, care and use of animals were followed. Throughout the duration of the study, animals were housed in small groups per cage. The stocking density of the cages was determined based on the recommendations from the German Society for Laboratory Animal Science, ensuring adequate space and welfare for the animals under study. The animals were housed in individually ventilated caging systems manufactured by Tecniplast, ensuring an environment with 14 hours of light and 10 hours of dark per day. Feed and bedding materials were sourced from an established provider certified under ISO9001 standards. Both materials underwent sterilization prior to use to maintain hygiene within the housing environment. Animals had *ad libitum* access to feed and water throughout the study, ensuring their needs were met without restriction. Environmental enrichment was provided in form of sterile bedding and paper tissues. Prior to the commencement of the experiments, all subjects underwent a visual stress assessment to evaluate their suitability for inclusion in the study. No animals had to be excluded due to presenting with phenotypic predispositions that could influence the outcomes. Mice were inspected minimum once daily, assessed and scored for disease activity using a pre-defined scoring sheet for tumor experiments as well as the body-conditioning score. Scoring methods had been developed in collaboration with the responsible veterinarian of the animal facility and had been preregistered and approved by the responsible government oversight committee. Stringent precautions were taken to minimize the risk of infection among the animals. Luminescence measurements were conducted under anesthesia to prevent the animals from experiencing pain. Animals meeting the predefined criteria for termination were euthanized using inhalation anesthesia followed by cervical dislocation to minimize suffering.

BLI analyses were done using the aura spectral instruments imaging software version 2.7.11 (XQuartz). Uniform regions of interest were used to analyze total flux (photon/s). For statistical analysis and plotting, GraphPadPrism 10.2.0 was used. Survival analyses were performed using the Kaplan-Meier method and Log-rank (Mantel cox) tests to compare treatment-groups. Differences with p < 0.05 (*), p < 0.01 (**) or p < 0.001 (***) were considered statistically significant.

**Immunohistochemistry of mouse organs**

For immunohistochemistry, organs were formalin-fixed and paraffin-embedded. Slices were incubated with a primary monoclonal mouse anti-human CD45 antibody (mouse monoclonal PD7/26 + 2B11, #ab781, Abcam) and a rabbit anti-GFP (polyclonal antibody, #A-11122, Invitrogen/ThermoFisher). Alexa Fluor 647-conjugated affiniPure F(ab‘)_2_ fragment goat anti-mouse IgG (#115-606-003, Jackson Immuno Research) and goat anti-rabbit IgG conjugated to Alexa Fluor 488 (#A-11008, Invitrogen/ThermoFisher) were used as secondary antibodies and nuclei were stained with 4’,6-Diamidin-2-phenylindol (DAPI) (Sigma-Aldrich). Pictures were taken with a BZ-X810 All-in-One Fluorescence Microscope (Keyence) with basic lenses (CFI PlanApo λ 10x 0.45/4.00 mm, CFI PlanApo λ 20x 0.75/1.00 mm) and filter cubes Ex: 360/40 DM: 400 BA: 460/50 (DAPI), Ex: 470/40 DM: 495 BA: 525/50 (Alexa Fluor 488) and Ex: 620/60 DM: 660 BA: 700/75 (Alexa Fluor 647).

**Primary patient material – histology**

Formalin-fixed paraffin-embedded (FFPE) tumor material was stained with hematoxylin and eosin (H+E) and immunohistochemically stained for ErbB2/HER2 (Dako). Chromogen in situ hybridization (CISH) was performed using the ZytoDot 2C SPEC ERBB2/CEN 17 probe (Cytovision).

# Supplementary Results


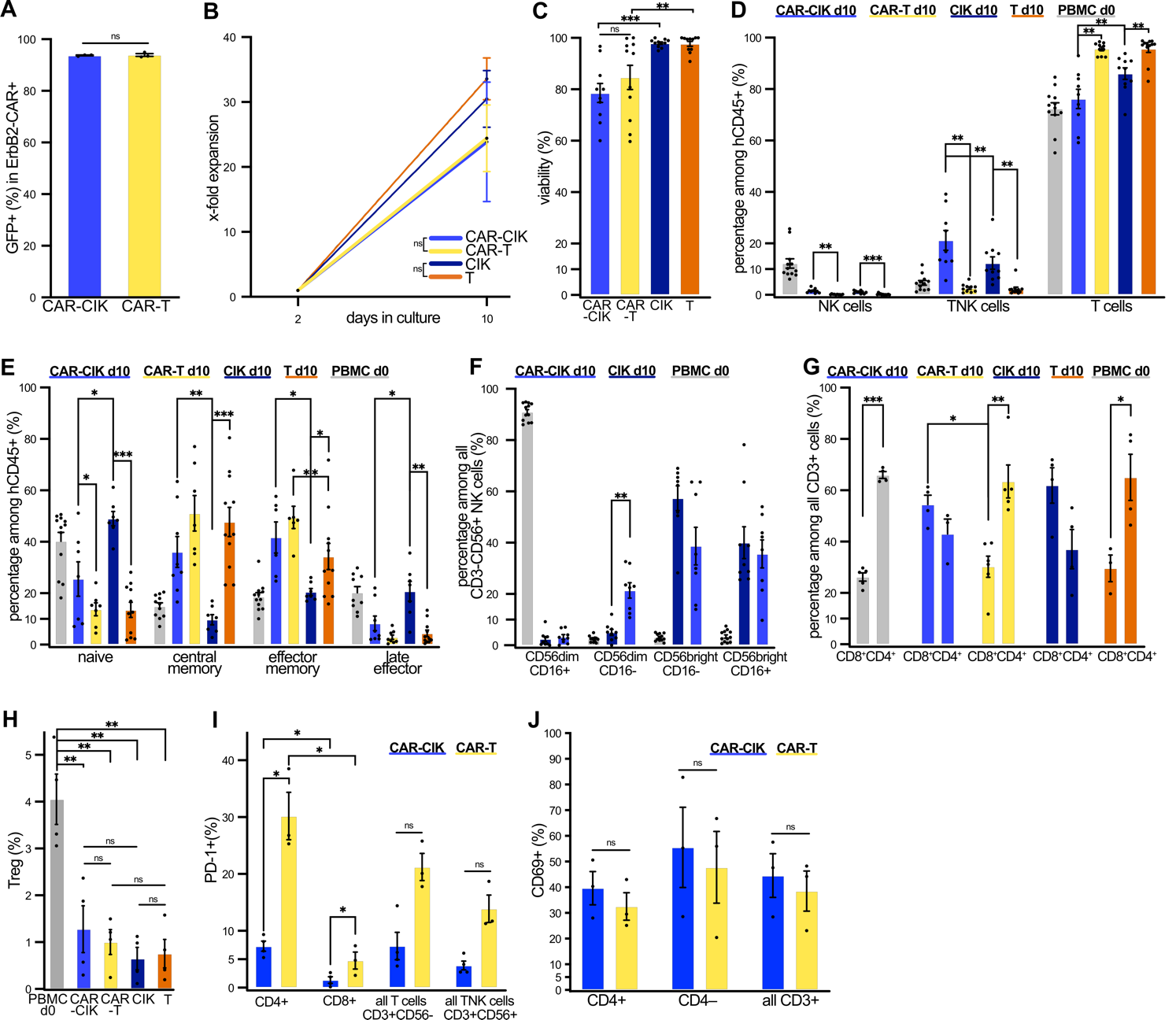


**Supplementary figure 1: Viability and phenotypic characterization of ErbB2-CAR-CIK and CAR-T cells after *in vitro* expansion.** Lentiviral transduction of CIK and T cells was performed on day 2 of culture, using a second-generation ErbB2-specific CD28-CD3ζ-CAR vector with an eGFP marker gene. All phenotypic endpoint characterizations were performed via flow cytometry on day +10 of *in vitro* expansion. **A)** Confirmation of GFP-coexpression on ErbB2-CAR+ cells. CAR expression was detected using an ErbB2-Fc-fusion protein (ErbB2-IgG-Fc chimera) as primary reagent and an anti-IgG-Fc as secondary antibody. Mean ± SEM of n=3. **B)** Growth of cells during cell culture, comparing fold expansion from day 2 to day 10. Mean ± SEM of n=8–13. P values by linear regression, comparing slope coefficients between CAR-CIK vs. CAR-T and CIK- vs. T-cells. **C)** Viable cells (percentage of total cells) on day 10 of culture assessed via trypan blue staining and manual counting using a hemacytometer. Mean ± SEM of n=10. **D)** Percentage NK cells (CD3-CD56+), TNK cells (CD3+CD56+) and T cells (CD3+CD56-) of starting material on day 0 (peripheral blood mononuclear cells, PBMC), untransduced CIK and T cells and CAR-CIK and CAR-T cells. Mean ± SEM of n=9–12. **E)** Subpopulations of naive (CD62L+CD45RO-), central memory (CD62L+CD45RO+), effector memory (CD62L-CD45RO+) and late effector (CD62L-CD45RO-) cells quantified among CAR-CIK and CAR-T cells, CIK and T cells. Mean ± SEM of n=6–12 experiments. **F)** Expression of CD56 and CD16 on CD3-CD56+ NK cells of source PBMCs on day 0 as well as CIK and CAR-CIK cells on day +10. Mean ± SEM of n=8–12. **G)** CD8+ and CD4+ phenotype of CD3+ cells in PBMC, CIK and T cells, CAR-CIK and CAR-T cells. Mean ± SEM of n=3–6. **H)** Percentage of regulatory T cells (Treg) of CD3+ cells, defined as CD3+CD4+CD25highCD127low. Mean ± SEM of n=4. **I, J)** Percentage of **(I)** PD-1+ cells and **(J)** CD69+ cells of indicated parental populations. Mean ± SEM of n=3. All P values (except B) by paired t-tests.

**
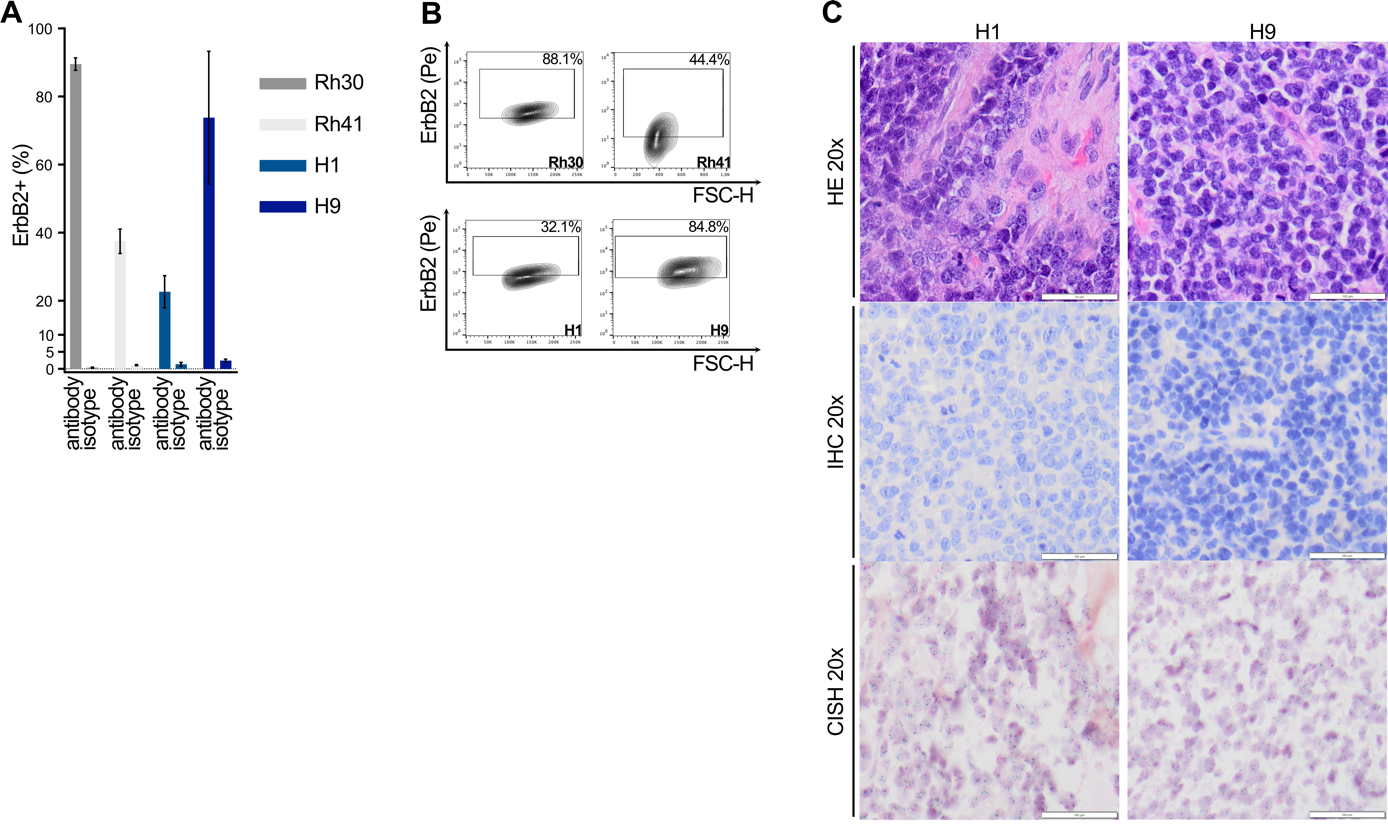
**

**Supplementary figure 2:** **ErbB2 expression in RMS cell lines and primary material. A)** Flow cytometric analysis of ErbB2 expression on RMS cells compared to isotype control for Rh30, Rh41 cell lines and primary tumor cells H1 and H9. Mean ± SEM of n=3 are shown. **B)** Representative flow cytometry plot of ErbB2 expression. **C)** Representative histology sections of primary patient samples H1 and H9 stained with hematoxylin and eosin (H+E), immunohistochemical (IHC) staining for ErbB2/HER2 and chromogen *in situ* hybridization (CISH) using the ZytoDot 2C SPEC ERBB2/CEN 17 probe, shown at 20-fold magnification. Scale bars indicate 100 µm.


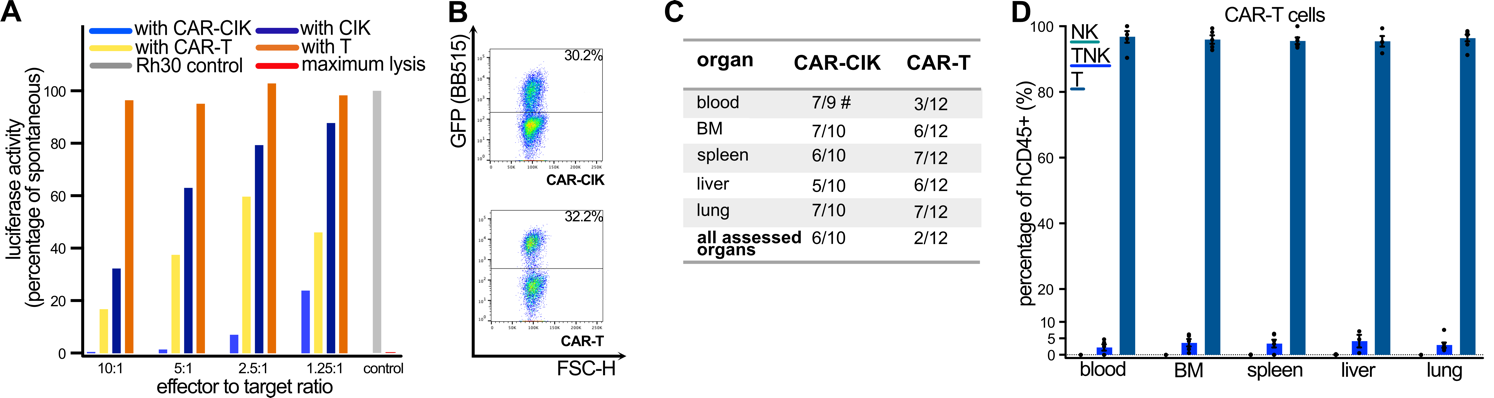


**Supplementary figure 3: Characterization of effector cells used for *in vivo* experiment and organ-wise itemization of hCD45+ effector cells A)** Cytotoxicity of effector cells used for *in vivo* experiments (related to figure 5 and 6). Co-incubation of parental and ErbB2-CAR CIK and T cells, or untransduced parental cells with Rh30Luc target cells for 24 h at different effector to target ratios. Results are given as percentage of luciferase signal of untreated Rh30Luc target cells. Triton X-100 treated cells served as maximum lysis control. **B)** CAR transduction measured via GFP expression in CAR-CIK and CAR-T cells used for *in vivo* experiments. **C)** Numbers of mice treated with CAR-CIK (n=10) or CAR-T cells (n=12) where human CD45+ cells were detected via flow cytometry in the indicated organs. (#: the blood sample was not available in one CAR-CIK treated animal.) **D)** TNK cell phenotype according to CD3 and CD56 expression of CAR-T cells harvested from indicated organs. Mean ± SEM of n=3–6.

**Supplementary table 1: Differentially expressed proteins in CAR-CIK vs. CAR-T cells**

Manual curation of differentially expressed proteins identified via whole cell proteomics in CAR-CIK vs. CAR-T cells (see figure 4). Proteins and respective genes, a short description of function and relevant literature are listed.

| Differential expression in CAR-CIK vs. CAR-T | Protein | Gene | Function |
| --- | --- | --- | --- |
| **Increased  expression in CAR-CIK** | Hemoglobine beta | HBB | INFy response (10)  ROS (reactive oxygen species) production linked to the production of granzymes and perforin (11) |
|  | Annexin A3 | ANXA3 | Th1 differentiation (12) Cytotoxic granule exocytosis in NK cells via STXBP2 (13) |
|  | Cathepsin H | CTSH | Effector molecule of granzyme B secretion in NK cells (14,15) |
|  | Cathepsin W | CTSW | Cellular cytotoxicity of NK cells and CD8+ T cells (16,17) |
|  | CD8a and  CD8b | CD8A CD8B | CD8+ T cell differentiation (18) |
|  | Neural Cell Adhesion Molecule 1 (CD56) | NCAM1 | NK cell marker (19) |
|  | T-box Transcription Factor or T-bet | TBX21 | Effector Tcell differentiation (20) IFNy response (21)  NK cell maturation and NK cell cytolytic activity (22) |
|  | Actin Filament Associated Protein 1 Like 2 | AFAP1L2 | Linked to activated and tumor-infiltrating NK cells (23) Linked to chronically stimulated T cells (24) |
|  | Ectonucleoside Triphosphate Diphosphohydrolase-1 | ENTPD1 | Linked to activated and tumor-infiltrating NK and T cells (25,26) Linked to chronically stimulated, exhausted T cells (27) |
|  | Syntaxin3 | STX3 | Granule exocytosis (28) Effector function of cytotoxic T cells (29) |
|  | T Cell Receptor Gamma Constant 1 | TRGC1 | Marker of gamma/delta T cells (30) Marker of NKT cells (31) |
| **Decreased expression in CAR-CIK** | Homer Scaffold Protein 2 | HOMER2 | Negative regulator of T cell activation (32) |
|  | Cytotoxic T-Lymphocyte Associated Protein 4 | CTLA4 | Negative regulator of T cell activation (33) Immunosuppressive function of regulatory T cells (34) |
|  | Legumain | LGMN | Induction of Th1 phenotype (35) Induction of effector memory phenotype in CD4+ T cells (35) Immunosuppressive function of regulatory T cells (36) |
|  | Hematopoietic Prostaglandin D Synthase | HPGDS | High expression in Th2 cells (37,38) |
|  | Glutathione Peroxidase 1 | GPX1 | Th2 differentiation (39) |
|  | Arginase 2 | ARG2 | Negative regulation of survival capacity of activated T cells (40)  Immunosuppressive function (of regulatory T cells) (41,42) |
|  | Monoaminoxidase A | MAO-A | Negative regulator of T cell function (43)  Associated with Th2 phenotype (12) |

**Supplementary References:**

1. Uphoff CC, Drexler HG. Detecting mycoplasma contamination in cell cultures by polymerase chain reaction. Methods Mol Biol Clifton NJ. 2011;731:93–103.

2. Zufferey R, Nagy D, Mandel RJ, Naldini L, Trono D. Multiply attenuated lentiviral vector achieves efficient gene delivery in vivo. Nat Biotechnol. 1997 Sep;15(9):871–5.

3. Abel T, El Filali E, Waern J, Schneider IC, Yuan Q, Münch RC, et al. Specific gene delivery to liver sinusoidal and artery endothelial cells. Blood. 2013 Sep 19;122(12):2030–8.

4. Koschade SE, Klann K, Shaid S, Vick B, Stratmann JA, Thölken M, et al. Translatome proteomics identifies autophagy as a resistance mechanism to on-target FLT3 inhibitors in acute myeloid leukemia. Leukemia. 2022 Oct 23;36(10):2396–407.

5. Heim C, Moser LM, Kreyenberg H, Bonig HB, Tonn T, Wels WS, et al. ErbB2 (HER2)-CAR-NK-92 cells for enhanced immunotherapy of metastatic fusion-driven alveolar rhabdomyosarcoma. Front Immunol. 2023 Aug 18;14(August):1228894.

6. McAlister GC, Nusinow DP, Jedrychowski MP, Wühr M, Huttlin EL, Erickson BK, et al. MultiNotch MS3 Enables Accurate, Sensitive, and Multiplexed Detection of Differential Expression across Cancer Cell Line Proteomes. Anal Chem. 2014 Jul 15;86(14):7150–8.

7. Zhu Y, Orre LM, Zhou Tran Y, Mermelekas G, Johansson HJ, Malyutina A, et al. DEqMS: A Method for Accurate Variance Estimation in Differential Protein Expression Analysis. Mol Cell Proteomics. 2020 Jun;19(6):1047–57.

8. Percie du Sert N, Hurst V, Ahluwalia A, Alam S, Avey MT, Baker M, et al. The ARRIVE guidelines 2.0: Updated guidelines for reporting animal research. Br J Pharmacol. 2020 Aug 14;177(16):3617–24.

9. Merker M, Wagner J, Kreyenberg H, Heim C, Moser LM, Wels WS, et al. ERBB2-CAR-Engineered Cytokine-Induced Killer Cells Exhibit Both CAR-Mediated and Innate Immunity Against High-Risk Rhabdomyosarcoma. Front Immunol. 2020 Oct 19;11(October):1–13.

10. Yang Q, Bai SY, Li LF, Li S, Zhang Y, Munir M, et al. Human Hemoglobin Subunit Beta Functions as a Pleiotropic Regulator of RIG-I/MDA5-Mediated Antiviral Innate Immune Responses. Williams BRG, editor. J Virol. 2019 Aug 15;93(16):1–21.

11. Morris G, Gevezova M, Sarafian V, Maes M. Redox regulation of the immune response. Cell Mol Immunol. 2022 Sep 2;19(10):1079–101.

12. Cano-Gamez E, Soskic B, Roumeliotis TI, So E, Smyth DJ, Baldrighi M, et al. Single-cell transcriptomics identifies an effectorness gradient shaping the response of CD4+ T cells to cytokines. Nat Commun. 2020 Apr 14;11(1):1801.

13. Li DQ, Lin M, Abdelrahman Z. High expression of the ANXA3 gene promotes immune infiltration and improves tumor prognosis in ovarian serous carcinoma using bioinformatics analyses. Ann Transl Med. 2022 Oct;10(19):1055–1055.

14. D’Angelo ME, Bird PI, Peters C, Reinheckel T, Trapani JA, Sutton VR. Cathepsin H Is an Additional Convertase of Pro-granzyme B. J Biol Chem. 2010 Jul;285(27):20514–9.

15. Magister Š, Tseng HC, Bui VT, Kos J, Jewett A. Regulation of split anergy in natural killer cells by inhibition of cathepsins C and H and cystatin F. Oncotarget. 2015 Sep 9;6(26):22310–27.

16. Wex T, Wex H, Hartig R, Wilhelmsen S, Malfertheiner P. Functional involvement of cathepsin W in the cytotoxic activity of NK‐92 cells. FEBS Lett. 2003 Sep 25;552(2–3):115–9.

17. Wex T, Bühling F, Wex H, Günther D, Malfertheiner P, Weber E, et al. Human Cathepsin W, a Cysteine Protease Predominantly Expressed in NK Cells, Is Mainly Localized in the Endoplasmic Reticulum. J Immunol. 2001 Aug 15;167(4):2172–8.

18. Kioussis D, Ellmeier W. Chromatin and CD4, CD8A and CD8B gene expression during thymic differentiation. Nat Rev Immunol. 2002 Dec 1;2(12):909–19.

19. Lanier LL, Testi R, Bindl J, Phillips JH. Identity of Leu-19 (CD56) leukocyte differentiation antigen and neural cell adhesion molecule. J Exp Med. 1989 Jun 1;169(6):2233–8.

20. Jaeger-Ruckstuhl CA, Lo Y, Fulton E, Waltner OG, Shabaneh TB, Simon S, et al. Signaling via a CD27-TRAF2-SHP-1 axis during naive T cell activation promotes memory-associated gene regulatory networks. Immunity. 2024 Feb;57(2):287-302.e12.

21. Szabo SJ, Kim ST, Costa GL, Zhang X, Fathman CG, Glimcher LH. A Novel Transcription Factor, T-bet, Directs Th1 Lineage Commitment. Cell. 2000 Mar;100(6):655–69.

22. Huang C, Bi J. Expression Regulation and Function of T-Bet in NK Cells. Front Immunol. 2021 Oct 5;12(October):1–9.

23. Canale FP, Neumann J, von Renesse J, Loggi E, Pecoraro M, Vogel I, et al. Proteomics of immune cells from liver tumors reveals immunotherapy targets. Cell Genomics. 2023 Jun;3(6):100331.

24. Good CR, Aznar MA, Kuramitsu S, Samareh P, Agarwal S, Donahue G, et al. An NK-like CAR T cell transition in CAR T cell dysfunction. Cell. 2021 Dec;184(25):6081-6100.e26.

25. Kang G, Zhao X, Sun J, Cheng C, Wang C, Tao L, et al. A2AR limits IL-15-induced generation of CD39+ NK cells with high cytotoxicity. Int Immunopharmacol. 2023 Jan;114(September 2022):109567.

26. Duhen T, Duhen R, Montler R, Moses J, Moudgil T, de Miranda NF, et al. Co-expression of CD39 and CD103 identifies tumor-reactive CD8 T cells in human solid tumors. Nat Commun. 2018 Jul 13;9(1):2724.

27. Moesta AK, Li XY, Smyth MJ. Targeting CD39 in cancer. Nat Rev Immunol. 2020 Dec 29;20(12):739–55.

28. Hackmann Y, Graham SC, Ehl S, Höning S, Lehmberg K, Aricò M, et al. Syntaxin binding mechanism and disease-causing mutations in Munc18-2. Proc Natl Acad Sci. 2013 Nov 19;110(47):E4482–91.

29. Pattu V, Qu B, Schwarz EC, Strauss B, Weins L, Bhat SS, et al. SNARE protein expression and localization in human cytotoxic T lymphocytes. Eur J Immunol. 2012 Feb;42(2):470–5.

30. Pizzolato G, Kaminski H, Tosolini M, Franchini DM, Pont F, Martins F, et al. Single-cell RNA sequencing unveils the shared and the distinct cytotoxic hallmarks of human TCRVδ1 and TCRVδ2 γδ T lymphocytes. Proc Natl Acad Sci. 2019 Jun 11;116(24):11906–15.

31. Fang Y, Bian C, Li Z, Jin L, Chen C, Miao Y, et al. ScRNA‐seq revealed disruption in CD8 + NKG2A + natural killer T cells in patients after liver transplantation and immunosuppressive therapy. Immun Inflamm Dis. 2023 Sep 27;11(9):1–14.

32. Huang GN, Huso DL, Bouyain S, Tu J, McCorkell KA, May MJ, et al. NFAT Binding and Regulation of T Cell Activation by the Cytoplasmic Scaffolding Homer Proteins. Science. 2008 Jan 25;319(5862):476–81.

33. Linsley PS, Brady W, Urnes M, Grosmaire LS, Damle NK, Ledbetter JA. CTLA-4 is a second receptor for the B cell activation antigen B7. J Exp Med. 1991 Sep 1;174(3):561–9.

34. Walker LSK. Treg and CTLA-4: Two intertwining pathways to immune tolerance. J Autoimmun. 2013 Sep;45:49–57.

35. Freeley S, Cardone J, Günther SC, West EE, Reinheckel T, Watts C, et al. Asparaginyl Endopeptidase (Legumain) Supports Human Th1 Induction via Cathepsin L-Mediated Intracellular C3 Activation. Front Immunol. 2018 Oct 24;9(October):1–7.

36. Probst‐Kepper M, Geffers R, Kröger A, Viegas N, Erck C, Hecht H ‐J., et al. GARP: a key receptor controlling FOXP3 in human regulatory T cells. J Cell Mol Med. 2009 Sep 29;13(9b):3343–57.

37. Mitson-Salazar A, Yin Y, Wansley DL, Young M, Bolan H, Arceo S, et al. Hematopoietic prostaglandin D synthase defines a proeosinophilic pathogenic effector human TH2 cell subpopulation with enhanced function. J Allergy Clin Immunol. 2016 Mar;137(3):907-918.e9.

38. Tanaka K, Ogawa K, Sugamura K, Nakamura M, Takano S, Nagata K. Cutting Edge: Differential Production of Prostaglandin D2 by Human Helper T Cell Subsets. J Immunol. 2000 Mar 1;164(5):2277–80.

39. Won HY, Sohn JH, Min HJ, Lee K, Woo HA, Ho YS, et al. Glutathione Peroxidase 1 Deficiency Attenuates Allergen-Induced Airway Inflammation by Suppressing Th2 and Th17 Cell Development. Antioxid Redox Signal. 2010 Sep;13(5):575–87.

40. Geiger R, Rieckmann JC, Wolf T, Basso C, Feng Y, Fuhrer T, et al. L-Arginine Modulates T Cell Metabolism and Enhances Survival and Anti-tumor Activity. Cell. 2016;167(3):829-842.e13.

41. Borek B, Gajda T, Golebiowski A, Blaszczyk R. Boronic acid-based arginase inhibitors in cancer immunotherapy. Bioorg Med Chem. 2020 Sep;28(18):115658.

42. Líndez AAM, Dunand-Sauthier I, Conti M, Gobet F, Núñez N, Hannich JT, et al. Mitochondrial arginase-2 is a cell‑autonomous regulator of CD8+ T cell function and antitumor efficacy. JCI Insight. 2019 Dec 19;4(24):1–21.

43. Wang X, Li B, Kim YJ, Wang YC, Li Z, Yu J, et al. Targeting monoamine oxidase A for T cell–based cancer immunotherapy. Sci Immunol. 2021 May 28;6(59):eabh2383.
